# Optimizing lipase production by *Bacillus subtilis* on cheese whey and evaluating its antimicrobial, antibiofilm, anti virulence and biosafety properties

**DOI:** 10.1038/s41598-025-92181-8

**Published:** 2025-04-01

**Authors:** Mohamed Y. Abo El-Naga, Muhammad A. Khan, Samah H. Abu-Hussien, Samar M. Mahdy, Ammar AL-Farga, Aml A. Hegazy

**Affiliations:** 1 Food Science Department, Faculty of Agriculture, Ain Shams University, Cairo, 11241 Egypt; 2 Department of Biological Sciences, Faculty of Sciences, International Islamic University (IIU), Islamabad, Pakistan; 3 Agricultural Microbiology Department, Faculty of Agriculture, Ain Shams University, Cairo, 11241 Egypt; 4 Department of Biochemistry, Faculty of Science, University of Jeddah, Jeddah, Saudi Arabia

**Keywords:** *Bacillus subtilis*, Lipase production, *Staphylococcus aureus*, Cheese whey, Antimicrobial activity, Molecular docking, Antimicrobials, Applied microbiology, Microbiology

## Abstract

This study optimized lipase production using cheese whey, biofilm inhibition, and antibacterial efficacy of *Bacillus subtilis* (DSM 1088)derived lipase against *Staphylococcus aureus* (ATCC 6538). Peak lipase activity, growth rate, and inhibitory potential were observed at 48 h and 30 °C. Using Plackett-Burman and Central Composite Designs (PBD and CCD), whey, peptone, and agitation speed were identified as significant factors, achieving optimal lipase activity of 1314 U/mL and an inhibitory zone diameter (IZD) of 48 mm against *S. aureus*. Partial purification through ammonium sulfate precipitation and dialysis increased partial purified lipase (PPL) activity by twofold and fivefold, respectively. PPL exhibited effective bactericidal properties with a minimum inhibitory concentration (MIC) and minimal bactericidal concentration (MBC) of 1/8 and 1/16, confirming a bactericidal effect (MIC/MBC ratio ≤ 2). Biofilm inhibition assays demonstrated 95% biofilm reduction at 80 µg/mL PPL, with SEM imaging revealing significant biofilm matrix disruption. Time-kill assays showed concentration-dependent bactericidal action, while inhibition of hemolysin and protease activities (25–100%) indicated reduced *S. aureus* pathogenicity. Cytotoxicity assays on normal liver cells showed an IC50 > 300 µg/mL, indicating low toxicity. GC/MS analysis of oil waste before degradation identified predominantly oleic acid 3-hydroxypropyl ester and octadecane derivatives, while after degradation, it revealed enriched free fatty acids including myristic, palmitic, linoleic, and oleic acids, which could enhance antimicrobial efficacy. Molecular docking suggested that PPL inhibits essential bacterial enzymes (folic acid synthetase, RNA polymerase, DNA gyrase), potentially disrupting DNA synthesis and promoting cell death. These findings highlight *B. subtilis*-derived lipase as a promising bio-agent for combating biofilm-associated, drug-resistant pathogens with clinical and industrial applications.

## Introduction

The global rise of multidrug-resistant (MDR) bacteria represents one of the most critical challenges in modern healthcare, demanding innovative approaches beyond traditional antibiotics. This growing threat has driven extensive research into alternative antimicrobial agents with novel mechanisms of action^1^. Among these, microbial enzymes have emerged as promising candidates, particularly for their ability to target bacterial structures through mechanisms distinct from conventional antibiotics^2^.

Lipases (triacylglycerol acyl hydrolases, EC 3.1.1.3) represent a significant class of microbial enzymes with demonstrated both antimicrobial and antibiofilm potentials^3^. These enzymes exhibit a distinctive mechanism of action against bacterial pathogens as they directly attack bacterial cell membrane integrity by hydrolyzing membrane lipids, leading to increased membrane permeability, causing cellular content leakage and ultimately cell death. This mechanism is particularly significant because it bypasses common antibiotic resistance mechanisms such as efflux pumps, target site modifications, and enzymatic inactivation. Additionally, lipases can degrade crucial bacterial cell wall components^4^, including lipopolysaccharides in Gram-negative bacteria and lipoteichoic acids in Gram-positive bacteria^5^, further enhancing their antimicrobial efficacy^3^, while also breaking down lipid components within the extracellular polymeric substances (EPS)^5^ matrix of biofilms. This dual action weakens both cellular and biofilm structural integrity, making bacteria more susceptible to antimicrobial treatments. Additionally, lipases interfere with bacterial communication by disrupting quorum sensing pathways^6^, which impairs biofilm development and virulence factor expression. The enzymes also modify the bacterial microenvironment by breaking down environmental lipids, thereby altering nutrient availability necessary for bacterial growth and biofilm formation^5^.

The sustainable production of microbial lipases aligns with environmental and economic goals, particularly through the utilization of agro-industrial byproducts like cheese whey. This nutrient-rich dairy byproduct serves as an ideal substrate for microbial growth and enzyme production, reducing industrial waste and supporting circular economy principles. Among various microbial sources, *B. subtilis* stands out for its efficient lipase production^7^ and demonstrated antimicrobial activity against *S. aureus*, a major MDR pathogen responsible for severe healthcare-associated infections.

To optimize lipase production and elucidate its antimicrobial mechanisms, this study integrates Response Surface Methodology (RSM)^8^ for efficient bioprocess optimization and molecular docking simulations to understand enzyme-target interactions^9^. RSM systematically evaluates interactions among multiple production parameters, while docking simulations provide insights into the molecular mechanisms underlying the antimicrobial activity of lipases.

Despite previous studies highlighting the antimicrobial potential of microbial lipases, significant knowledge gaps remain regarding production optimization, specific enzyme-pathogen interactions, and their application in combating MDR pathogens. This study addresses these gaps by employing a multidisciplinary approach combining sustainable production practices, bioprocess optimization, and computational analysis.

The present study aims to optimize *B. subtilis* lipase production using cheese whey as a substrate, evaluate its antimicrobial, antibiofilm, and anti-virulence activities against *S. aureus*, and assess its cytotoxicity profile against normal liver cells. The findings are expected to contribute to developing novel strategies against antibiotic-resistant pathogens while promoting sustainable biotechnology practices.

## Materials and methods

### Whey powder

Whey powder (commercial spray dried), sourced from Maybi, Turkey (https://www.maybi.com.tr/), was utilized as the growth medium for *B. subtilis* to produce lipase. The powder’s average composition included 11.2% protein, 0.9% fat, 4% moisture, 70% lactose, and 10.9% ash^10^.

### Chemicals

Oleic acid was purchased from Sigma–Aldrich (USA). Olive oil was obtained from commercial sources. Cupric acetate, isooctane, and acetone were purchased from Merck Limited (Mumbai, India). All other chemicals and reagents used were of analytical grade. Copper reagent was prepared according to Kwon and Rhee^11^ Briefly, a 5% (w/v) aqueous solution of cupric acetate was prepared, and, after filtration, pH was adjusted to 6.1 using pyridine. All microbiological nutrient media were obtained from (Oxoid, Basingstoke, England).

### Collection of frying oil waste

Frying oil waste samples were collected from local restaurants in Cairo, Egypt. All samples were transported to Microbial Resources Center (MIRCEN) lab. Located in the Faculty of Agriculture, Ain Shams University. The frying oil waste is cooled to room temperature, then centrifuged at 10,000 rpm for 15 min to separate food particles, water and solids. All treated oil samples were transferred to glass containers for storage in cool and dark conditions for further studies.

### Microorganisms and media used

*S. aureus* (ATCC 6538) and *B. subtilis* (DSM 1088) strains were obtained from MIRCEN in Cairo, Egypt. The Culture was maintained on Tryptic soy medium slants at 4 °C and sub-cultured at monthly intervals. Tryptic soy Medium Base agar and broth were used to cultivate, purify and maintain *B*. *subtilis*^12^. To prepare whey medium: whey 3% (w/v) was prepared using distilled water and supplemented with 2% olive oil (v/v). pH was adjusted to 7 ± 0.2 using NaOH (0.1 N). supplemented with 2% (v/v) frying oil waste was used to detect the optimal incubation time for initial lipase production. Tryptic soy broth (g/L) (pancreatic casein 17, K_2_HPO_4_ 2.5, glucose 2.5, NaCL 5 ,peptone 3) was used for *S*. *aureus* inoculum preparation and maintenance. Muller Henton agar (MHA) medium^13^ was used to test the inhibitory potential of crude lipase against *S. aureus* expressed as inhibition zone diameter (IZD) in whey. All prepared media were sterilized by autoclaving at 121℃ for 15 min.

### Preparation of standard inoculum

For the preparation of standard inoculum, 50 ml of Tryptic soy broth medium were prepared in 250 mL Erlenmeyer flasks and inoculated with a full loop of cultivated slants. Incubation of the flasks was carried out using a rotary shaking incubator (Lab-line Ltd.) at the rate of 120 rpm for 24 h at 30 °C and considered as the standard inoculum (1.0 × 10^7^/mL viable cells) for shake flasks experiments.

### Time course of lipase production

Whey medium, supplemented with 2% sterilized frying oil waste, was used as the lipase production medium to study the time course of enzyme production. A 5% inoculum was added to 50 mL of medium, in 250-mL Erlenmeyer flasks and incubated at 150 rpm on a rotary shaker, at 37 °C, for 96 h. Samples were collected periodically at 24 h intervals. Cell dry weight, lipase activity, and inhibition zone of *S. aureus* were determined as described later. All experiments were done in triplicate. The logarithmic phase regression coefficient was estimated based on the correlation between time (h) and growth rate (µ), lipase activity (U/mL), and inhibition activity (IZD) in millimeters. Lipase activity was determined as described later. 

The specific growth rate (µ/h) was calculated as follows:

1 $$Specific~growth~rate~\left( {\mu /h} \right)~ = \frac{{\left( {\ln ~X{-}\ln ~X0} \right)~}}{{~\left( {t~ - ~t0} \right)~~}}$$

At the logarithmic phase, doubling time (td) and multiplication rate (MR) were calculated as follows:

2 $$td~=\frac{{\ln 2~}}{{\mu g~}}$$

3 $$MR=\frac{{1~}}{{td~}}$$

### Screening and optimization of the most significant parameters affecting lipase production by *B*. *subtilis* in submerged culture

#### Plackett-burman design (PBD)

Plackett-Burman Design (PBD)^8^ was used to screen and evaluate the impact of different nutritional and physical requirements for lipase production by *B. subtilis*. The experimental Plackett–Burman design was analyzed using “Design Expert^®^ 11” (Stat-Ease, Inc., Minneapolis, USA). A total of 8 variables were selected for the study in 12 experiments with each variable being represented at two levels, high (+ 1) and low (-1) as presented in Table S1. The main variables selected for the present study were frying oil waste (mL/L), whey (mL/L), tryptone (g/L), pH, MgSO_4_ (g/L), peptone (g/L), mannitol (g/L) and agitation speed (rpm). All media were inoculated with 5% of standard inoculum (1 × 10^7^ CFU/mL). After inoculation, flasks were incubated in a shaker incubator (Lab-line Ltd.) at 30^°^C with shaking at different agitation speed rates according to the run number. Based on the effect of incubation time on lipase activity results, lipase production was measured after 48 h of incubation. CDW (g/L), Lipase activity (U/mL), Total protein (mg/mL), Specific activity (U/mg), and IZD (mm) of *S. aureus* were determined^14^.

#### Central composite design (CCD) for lipase production optimization by *B. subtilis*

A central composite design (CCD)^15^ was applied for the second optimization step in lipase production by *B. subtilis*, focusing on three significant variables from the PBD design: whey, peptone, and agitation speed as presented in Table S2. These variables were tested at three levels (-1, 0, + 1) in a 20-run experiment. Key responses such as the cell dry weight (CDW), lipase activity, total protein content, specific activity, and inhibition zone diameter (IZD) were measured. A second-order polynomial model was fitted to the data using the following equation: Y = β0 + β1A + β2B + β 11A^2^ + β 22B^2^ + β 12AB in which, Y, predicted response; β0 intercept; β1, β2, linear coefficients; β11, β22, squared coefficient; β12 as an interaction coefficient. ANOVA was carried out to indicate the model’s fit. Contour and 3D surface plots were created to show the relationships between the experimental factors and responses^16^. Assumptions of normality and homogeneity of variances were assessed using diagnostic tools within Design-Expert 11 software, including normal probability plots of residuals and residuals against. predicted plots.

#### Partial purification of lipases produced by *B. subtilis*

For lipase purification, the crude lipase was obtained from the most effective run according to the results of CCD design to be purified. Briefly, solid ammonium sulfate (40–80% saturation) was added as per standard chart to precipitate out the enzyme. Precipitation was done at 4ºC. The precipitate was collected by centrifugation (10000 rpm, 4ºC for 15 min.) and re-suspended in 0.9% NaCl and then dialyzed against phosphate buffer at 4ºC to prevent contamination of the final preparation before dialysis. The dialyzed fraction was made up and referred to as partially purified lipases. The activity of partially purified lipases was assayed as described later. The partially purified enzyme (PPL) was di-filtered using Amicon UF Stirred Cell (Model 8010) with a 10 kDa cut-off membrane against Tris-HCl buffer (pH 8.5) to remove ammonium sulfate^17^.

### Lipase activity assay

Lipase activity was determined using the copper soap method^18^. The substrate consisted of 1.25 ml of emulsion of 2% polyvinyl alcohol–olive oil (3:1 ratio) and 1.25 ml of phosphate buffer (pH 6.5), preincubated for 10 min at 37 °C. Then, 2.5 mL of *B. subtilis* culture containing crude enzyme or 50 mg of PPL were added and mixed vigorously for 1 min with magnetic stirring (500 rpm). The enzymatic reaction was initiated by adding olive oil (10% v/v), and 400 µL aliquots were collected at predetermined intervals. The reaction was stopped with 5 ml of acetone followed by 2.5 ml of isooctane. The resultant mixture was vortexed and boiled for 1 min. Each collected sample was diluted with 4.6 mL of isooctane or benzene, mixed vigorously for 30 s, and treated with 1 mL cupric acetate reagent. After vortex-mixing for 30 s and centrifugation at 10,000 rpm for 5 min, the absorbance of the colored upper layer containing free fatty acids (FFAs) was measured at 715 nm using a Shimadzu 2401 UV–VIS spectrophotometer. Controls were prepared by adding 2.5 mL of *B. subtilis* culture containing crude enzyme or 0.1mL of PPL after stopping the reaction. One unit of lipase activity (U) was expressed as µmol free fatty acid released/1mL culture or 1mL of PPL/min at 37 °C, calculated by subtracting control readings from sample readings. FFAs were quantified using a standard curve prepared with oleic acid (0–1000 µmol) in 2.5 ml of isooctane and 1 ml of copper reagent.

### Protein determination

The total protein was determined using bovine serum albumin (BSA) as standard solution^14^. Total protein was calculated to estimate the specific activity of lipase enzyme (U/mg) as follows:

4 $$Lipase~Specific~activity~~=\frac{{~Lipase~activity}}{{Total~protein~}}$$

### Inhibitory activity of PPL produced by *B. subtilis* against *S. aureus*

Standard guidelines of (Clinical Laboratory Standards Institute) CLSI^19^ were carried out using micro-diffusion assay. Briefly, Muller solidified Hinton agar plates were inoculated with 0.1mL of *S. aureus* strain standard inoculate by spreading technique. Wells were made using a sterilized cork borer (6 mm in diameter) and filled with 10µL of PPL against a control well filled with ampicillin. Thereafter, plates were incubated at 30℃ for 24 h. All trials were carried out in triplicates. IZD was measured and recorded.

### Minimum inhibitory concentration (MIC) of PPL against *S. aureus*

*S. aureus* strain inoculum was added to tubes containing MHB medium with PPL in serial twofold dilutions (0 (control), 1/2, 1/4, 1/8, 1/16). The control tube was free from PPL. All the tubes were incubated at 37 °C for 24 h. Optical density (O.D) at 620 nm was measured. MIC is defined as the lowest concentration of PPL that inhibits the growth of *S. aureus*. All trials were carried out in triplicate. For Minimum bactericidal concentration (MBC) calculation, negative results of the MIC test were subcultured on MHA medium and incubated at 37 °C for 24 h. The lowest concentration of PPL that showed no growth on the MHA medium was recorded as the MBC value. All trials were carried out in triplicate. Based on MIC, MBC results. MBC/MIC ratio was calculated^20^. The PPL mode of action was assessed according to the following equation:

5 $$~PPL~Mode~of~action=\frac{{MBC~value}}{{MIC~value}}$$

PPL is considered a bactericidal agent if the MBC/MIC value is ≥ 4. On the other hand, it is considered bacteriostatic if the value is ≤ 2.

### Biofilm Inhibition of PPL against *S. aureus*

The biofilm formation assay was conducted by growing the strain culture in test tubes with nutrient broth supplemented with mannitol for 24 h at 37 °C. After incubation, the culture was removed, test tubes were washed with phosphate-buffered saline (PBS), and the remaining biofilm was stained with crystal violet solution. The excess stain was washed off, and the bound stain was solubilized using acetic acid. The biofilm was quantified by measuring the absorbance at 595 nm. Biofilm eradication was calculated as [OD control - OD test)/OD control) x 100]. For microscopic analysis, biofilms were grown on glass coverslips in 6-well plates using *S. aureus* (10^6^ CFU/mL in nutrient broth with 1% mannitol) for 48 h. After PPL treatment (10, 20, and 40 µg/ml) for 24 h, coverslips were stained with crystal violet and examined at 100X magnification^21^. All trials were carried out in triplicate.

### Scanning Electron microscope (SEM) analysis

The antibiofilm potential of the produced PPL enzyme on preformed *S. aureus* biofilms was visualized using scanning electron microscopy (SEM), following a protocol adapted from a previous study^5^.

### Time kill assay of PPL against *S. aureus*

*S. aureus* was cultured in LB broth (initial OD 600 = 0.3) with PPL at 3/4, 1/2, and 1/4 MIC. Growth was monitored spectrophotometrically (OD 600) at 30-min intervals for 120 min at 37 °C. Control cultures without PPL were maintained under identical conditions. All trials were carried out in triplicate .

### Effect of PPL on protease and hemolysin production

Overnight culture of the tested *S. aureus* was grown in Luria-Bertani (LB) broth, with and without supplementation of the produced PPL enzyme at concentrations of 2 and 4 µg/mL. The cultures were streaked into skim milk agar plates and human sheep agar plates, respectively. After incubation at 37 °C for 24 h, the formation of clearance zones around the wells was observed, indicating the presence of proteolytic activity and hemolysis of red blood cells^22^. The diameters of these clear zones were measured to assess the proteolytic potential of the supernatants obtained from cultures grown in the presence and absence of the PPL enzyme. All trials were carried out in triplicate.

### Cytotoxicity of PPL against normal mice liver cell line

The cytotoxicity of the produced PPL was evaluated at Nawah Scientific (https://nawah-scientific.com/) located in Cairo, Egypt using an MTT assay. Suspension cells in a 96-well plate were centrifuged, and media was carefully aspirated. Each well received 50 µl of serum-free medium and 50 µl of MTT solution, then incubated at 37 °C for 3 h. Mouse liver cells were cultured in RPMI medium with 10% FBS, antibiotics, and 5% CO_2_. For the assay, cells were treated with various PPL concentrations. After 48 and 72 h, MTT solution was added, incubated, and the resulting formazan crystals were dissolved in DMSO. Absorbance was measured at 570 nm using an ELISA plate reader^23^. Cell viability (%) and cytotoxicity (%) were calculated according to the following equations:

6 $${\text{Cell viability~}}\left( {\text{\%}} \right)=\frac{{{\text{Absorbance of treated cells}}}}{{{\text{Absorbance of control cells}}}} \times 100$$

7 $${\text{Cytotoxicity}}\left( {\text{\%}} \right)=100 - {\text{Cell viability }}\left( {\text{\%}}\right)$$

Where: Absorbance is measured at 570 nm, Control cells are untreated cells, treated cells are exposed to different PPL.

### Gas chromotography mass spectrometry (GC/MS) for the frying oil waste and the produced metabolites by *B. subtilis* grown on Whey

Before the GC/MS assay, the samples described above were further concentrated by evaporation and then dissolved into methanol. Further, the extract was analyzed in GC/MS to investigate the intermediate products produced during the biodegradation of whey by *B. subtilis*. The chemical composition was performed using a Trace GC/TSQ mass spectrometer (Thermo Scientific, Austin, TX, USA) with a direct capillary column TG–5MS (30 m x 0.25 mm x 0.25 μm film thickness). The column oven temperature was initially held at 50 °C and then increased by 5 °C /min to 250 °C held for 2 min increased to the final temperature 300 °C by 30 °C /min and held for 2 min. The injector and MS transfer line temperatures were kept at 270, and 260 °C respectively; Helium was used as a carrier gas at a constant flow rate of 1 ml/min. The solvent delay was 4 min and diluted samples of 1 µl were injected automatically using Autosampler AS1300 coupled with GC in the split mode. EI mass spectra were collected at 70 eV ionization voltages over the range of m/z 50–650 in full scan mode. The ion source temperature was set at 200 °C. The components were identified by comparison of their mass spectra with those of WILEY 09 and NIST 14 mass spectral database^24^.

### Molecular Docking

Protein structures for all the molecules involved in molecular docking were obtained from UniProtKB using the following protein identifiers: gryA (A0A448BHV3), gryB (A0A172WC02), folA (Q4K492), folP (Q4KIG1), Blact (Q00626), PPL (C0KTV1), and RNAp (I3NRM6). Subsequently, these structures were submitted to the Deepsite PlayMolecule platform (https://www.playmolecule.com/deepsite/) to identify the amino acids constituting the pocket binding sites. All ligands’ SMILES and SDF (structure data files) were retrieved from the PubChem database (https://pubchem.ncbi.nlm.nih.gov/). The receptor was prepared using Autodock tools v4.2.6 (https://vina.scripps.edu/ ). Docking simulations were performed using Autodock Vina v1.1.2 (https://vina.scripps.edu/ ), with a grid box size of 20 × 20 × 20 ^9^.

### Statistical analysis

ANOVA was performed using Design-Expert 11 software (www.statease.com ). Post-hoc multiple comparisons were conducted using Tukey’s HSD to determine significant differences between group means. For shake flask experiment trials, a comparison of three replicates’ mean was compared using SPSS 26.0 at a significance level of *p* < 0.05 ^25^.

Assumptions of normality and homogeneity of variances were assessed using diagnostic tools within Design-Expert, including normal probability plots of residuals and residuals vs. predicted plots.

## Results

### Effect of incubation time on the growth rate, lipase activity, and inhibitory potential of *B. subtilis*

**Fig. 1:**
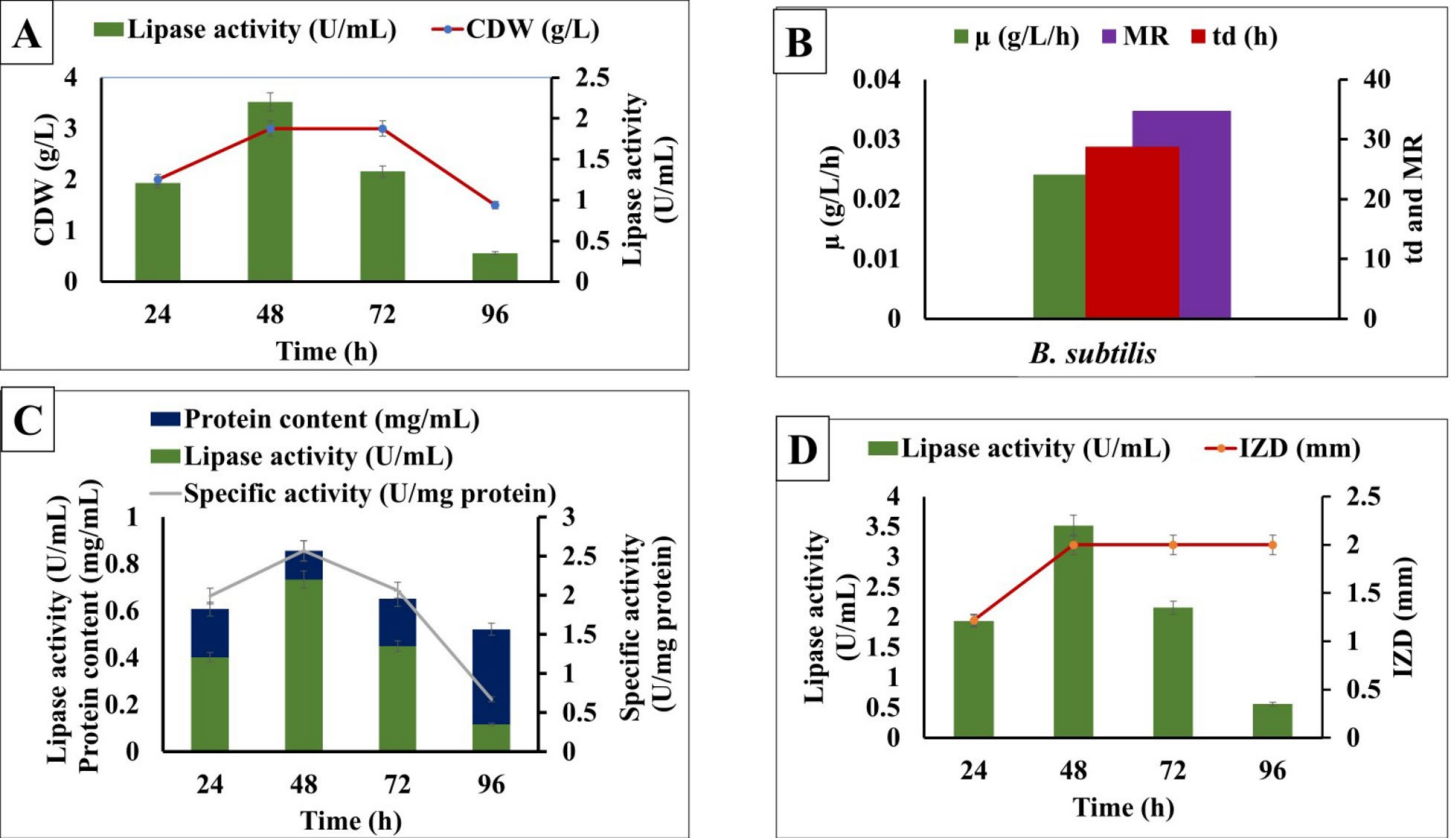
Effect of incubation time on cell dry weight (g), lipase activity (U/mL), and inhibition zone diameter (mm) against *S. aureus* from *B. subtilis* grown on whey medium.

The amount of lipase activity was observed during 96 h of incubation at 30℃. The maximum growth rate, lipase activity, and inhibitory potential were observed after 48 h of incubation. Growth rate (µ) was 0.023 g/L, doubling time (td) and multiplication rate (MR) were 29.4 h and 0.03 respectively, for the *B. subtilis* strain as presented in Fig. 1. At longer incubation periods, the lipase activity decreased. A significant correlation between incubation time against growth rate, lipase activity, and inhibitory potential was observed with correlation coefficients (R^2^) of 0.97, 0.98, and 0.97, respectively. Growth kinetics for the logarithmic phase revealed that the specific growth rate, specific lipase activity rate, and specific inhibition rate were 0.022h^-1^ , 0.025 (U/mg protein/h), 0.021 (mm/h), respectively.

### Optimization of PPL using response surface methodology (RSM)

#### Plackett- Burman design (PBD) for screening variables affecting lipase production by *B. subtilis*

**Fig. 2:**
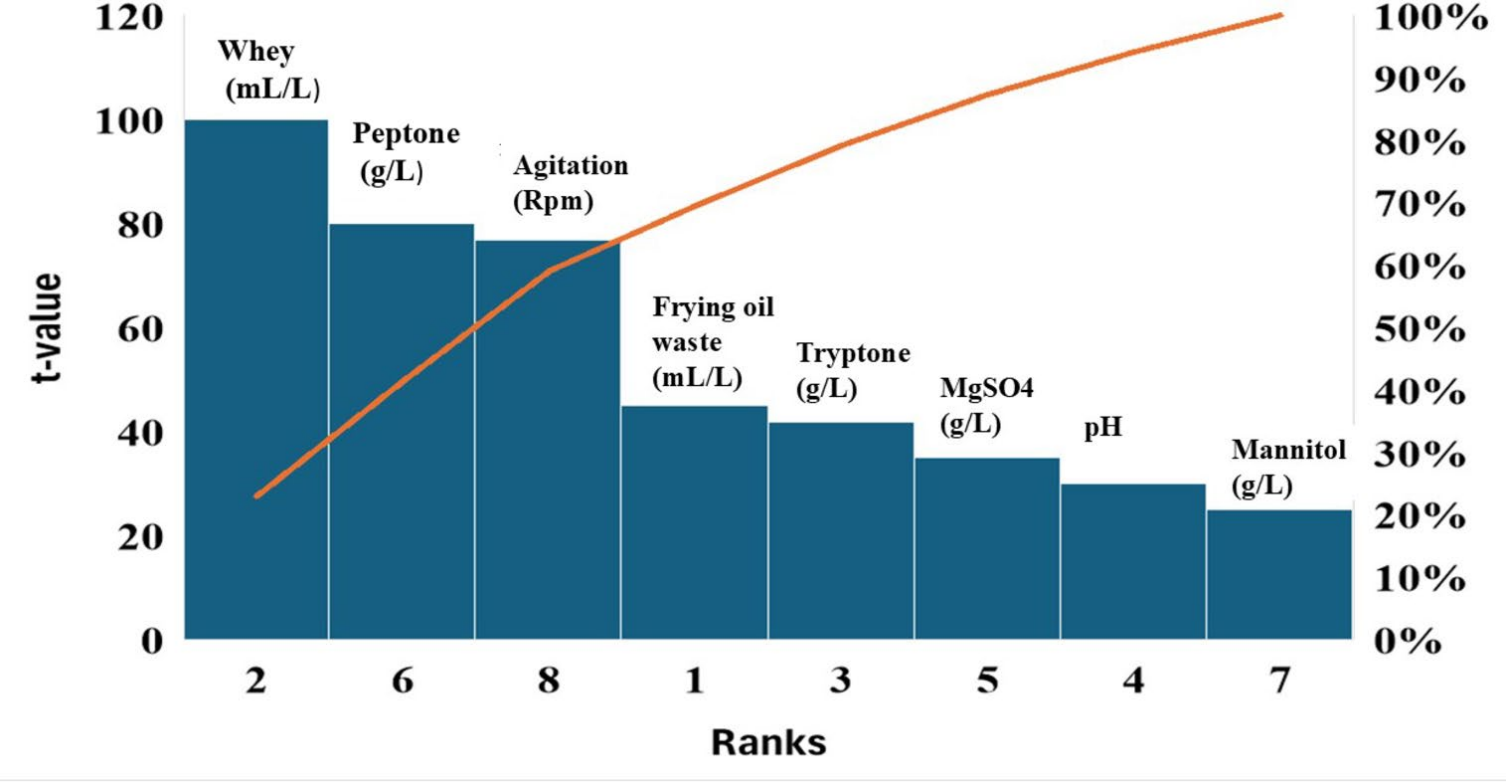
Effects of different factors tested in Plackett–Burman design for lipase production from *B. subtilis*. The main effects plot of PBD indicates that B: whey (g/L), E: peptone (g/L), and E: agitation (rpm) are the main significant factors affecting lipase production (U/mL) and IZD (mm) of *B. subtilis* against *S. aureus.*

The PBD design was carried out in 12 trial runs with high and low levels for each variable to detect the main significant factors in lipase production. Eight factors, namely frying oil waste (A), whey (B), tryptone (C), pH (D), MgSO_4_ (E), peptone (F), mannitol (G), and agitation speed (H) were analyzed by PBD as illustrated in Fig. 2 which showed the conjugation between the main effects and ANOVA analysis to determine the level mean differences for all factors. In the present study, whey, peptone, and agitation speed affected lipase production in their high levels at 40 mL/L, 30 g/L, and 250 rpm, respectively.

### Central composite design (CCD) for lipase production optimization by *B. subtilis*

**Fig. 3:**
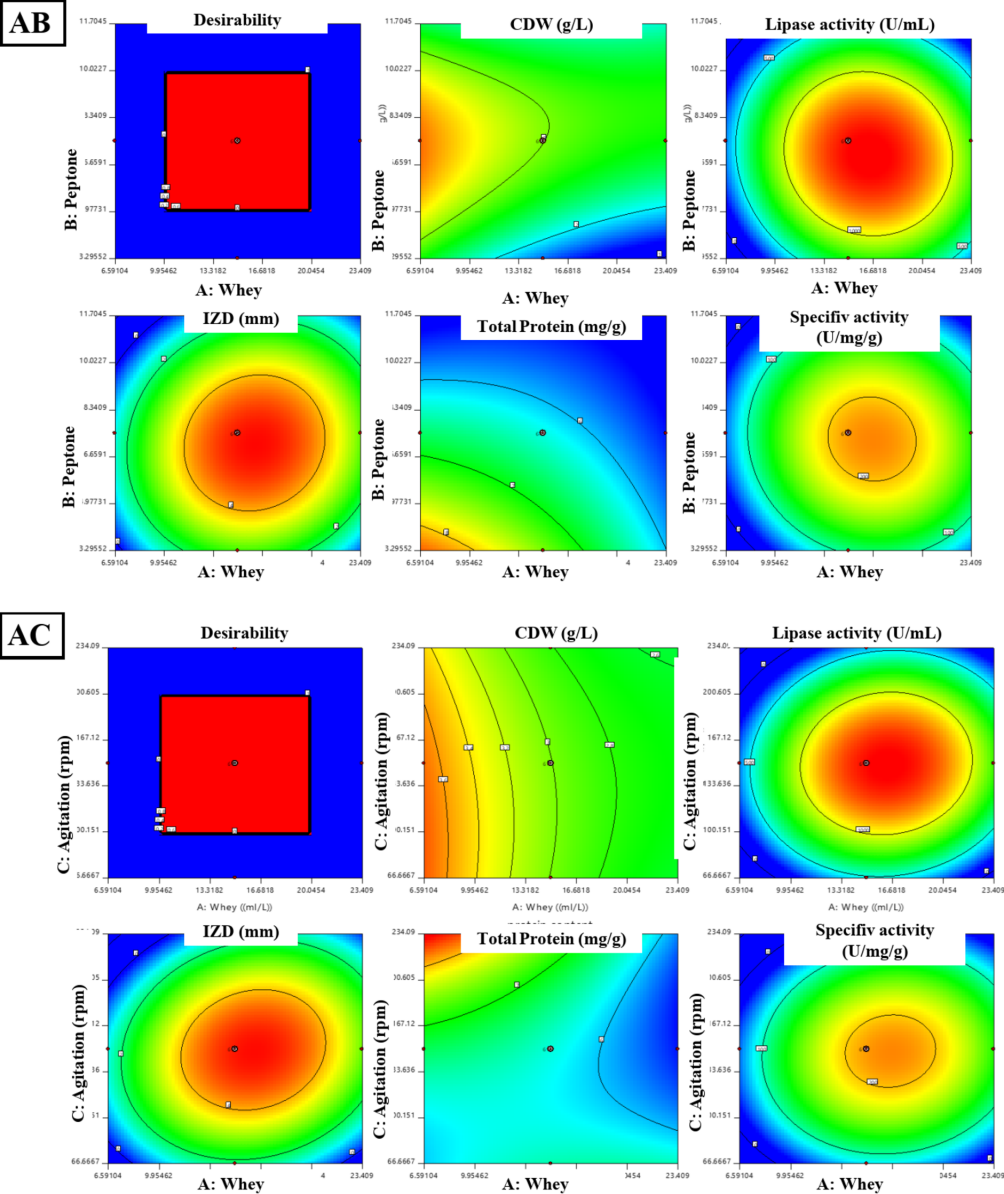

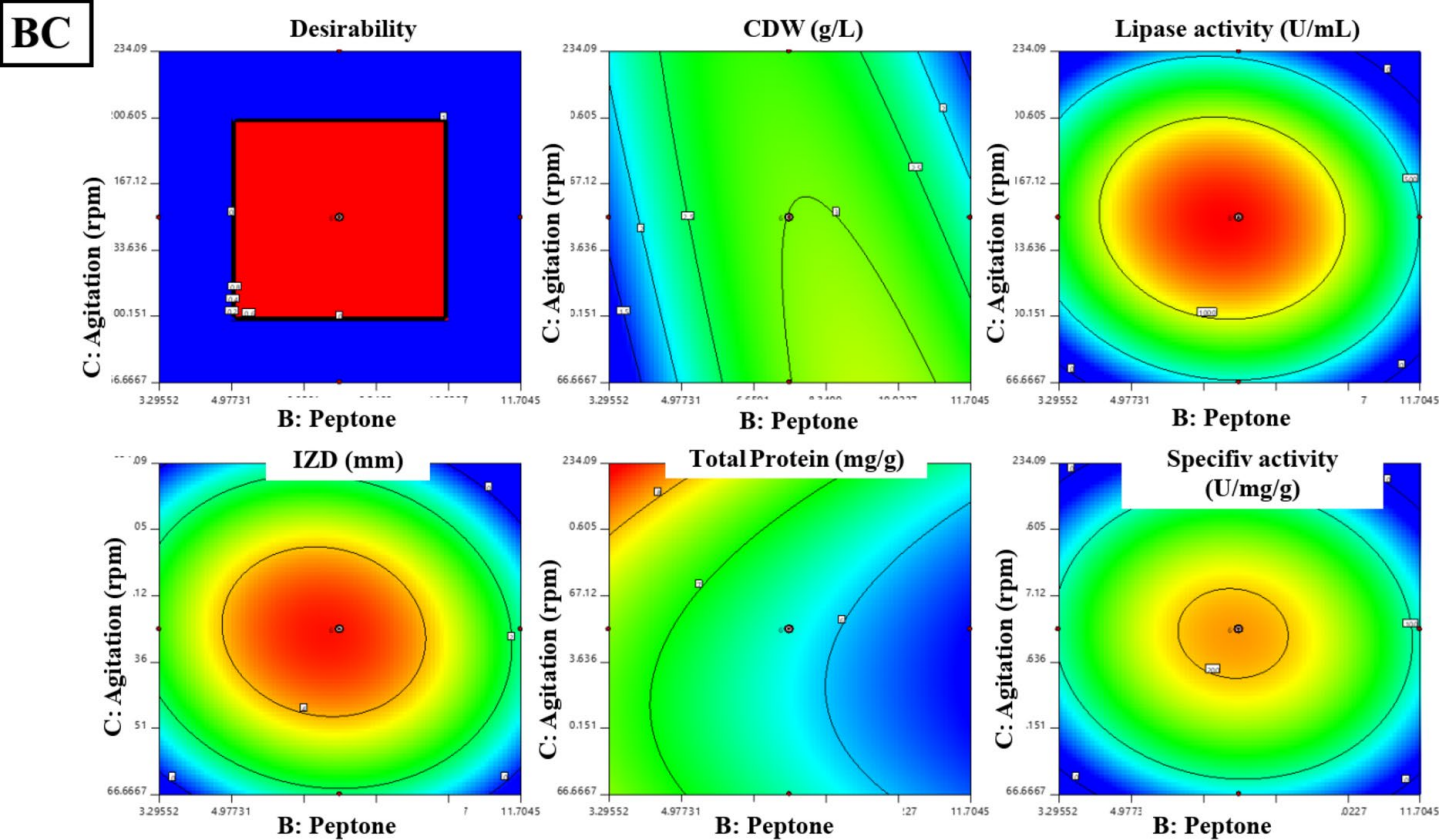
The 2d contour lines for the interaction between the three media components (whey, peptone, and agitation speed) on CDW (g/L), lipase activity (U/mL), and IZD (mm) against *S. aureus*. AB: effect of whey (g/L) and peptone (g/L) on CDW (g/L), lipase activity (U/mL). AC: effect of whey (g/L) and agitation (rpm) on CDW (g/L), lipase activity (U/mL) and IZD (mm) against *S. aureus.* BC: effect of peptone (g/L) and agitation (rpm) on CDW (g/L), lipase activity (U/mL), and IZD (mm) against *S. aureus.*

**Fig. 4:**
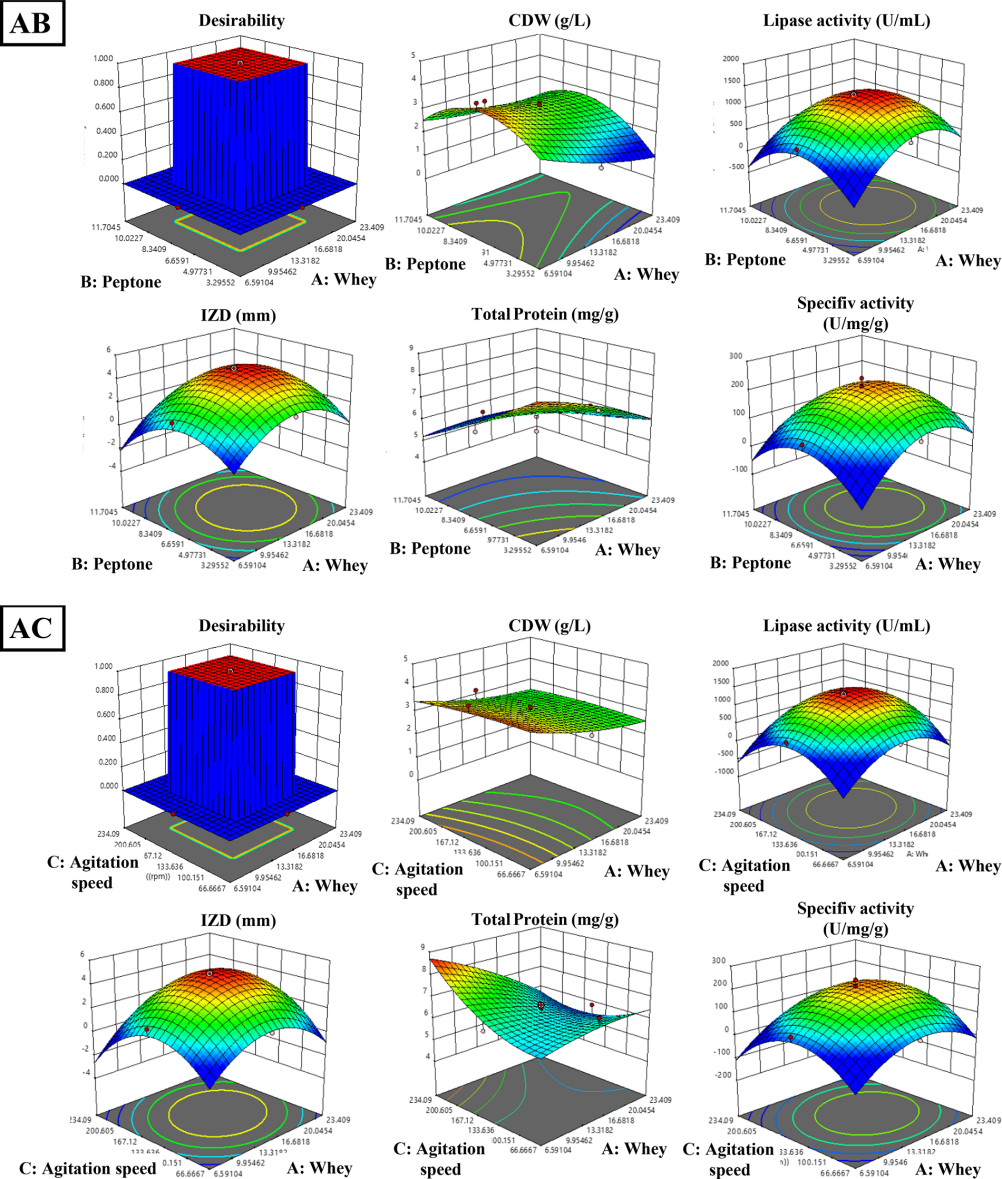

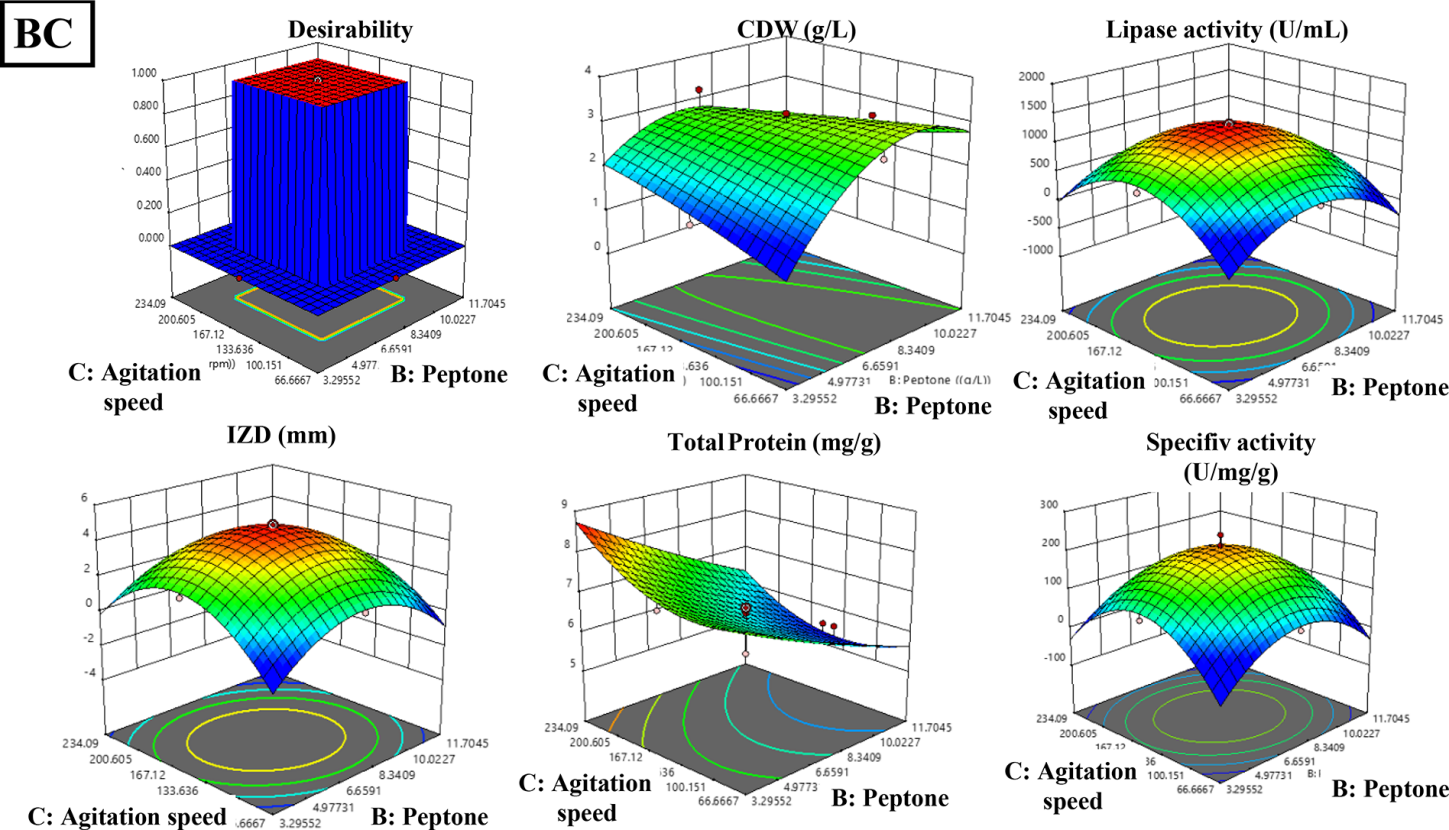
3D graphs for the interaction between the three media components (whey, peptone, and agitation speed) on CDW (g/L), lipase activity (U/mL), and IZD (mm) against *S. aureus*. AB: effect of whey(g/L) and peptone (g/L) on CDW (g/L), lipase activity (U/mL). AC: effect of whey (g/L) and agitation (rpm) on CDW (g/L), lipase activity (U/mL) and IZD (mm) against *S. aureus.* BC: effect of peptone (g/L) and agitation (rpm) on CDW (g/L), lipase activity (U/mL), and IZD (mm) against *S. aureus.*

The optimization of lipase production and its anti-staphylococcal activity from *B. subtilis* was achieved using central composite design (CCD) and illustrated in Table 1, revealing significant models for all responses. The model for cell dry weight (F = 4.71, *P* = 0.0019, R²=0.80) showed notable interactions between peptone-whey and peptone-agitation speed. Lipase activity demonstrated a model (F = 19.4, *P* < 0.0001, R²=0.94) significantly influenced by whey, peptone, and their quadratic effects, achieving 1318 U/mL experimentally against 1314 U/mL predicted. The inhibitory activity against *S. aureus* exhibited similar statistical strength (F = 18.92, *P* < 0.0001, R²=0.94), with observed IZD (48 mm) closely matching predicted values (49 mm). Protein content (F = 5.03, *P* = 0.0094, R²=0.81) was directly affected by all three variables without significant interactions, while specific lipase activity (F = 13.33, *P* = 0.0002, R²=0.92) was primarily influenced by whey concentration and quadratic effects of all variables. Three-dimensional response surfaces and contour plots in Figs. 3 and 4 illustrate the optimal conditions for maximum lipase production and antistaphylococcal activity, demonstrating the successful optimization of process parameters through RSM.

**Fig. 5:**
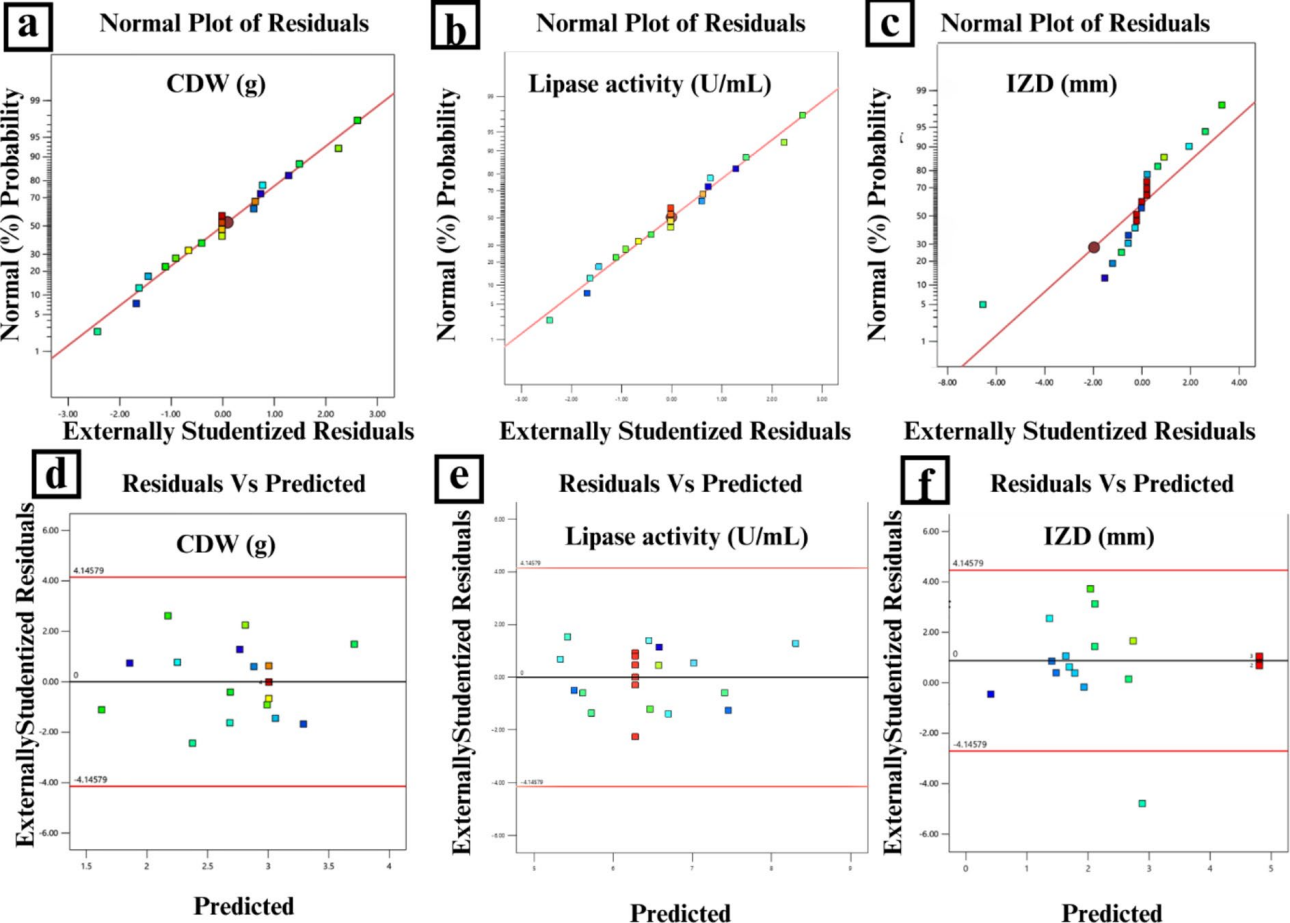
Normal probability plots and residual analyses for optimization of lipase production. (**a**–**c**) Normal probability plots of externally studentized residuals showing the distribution of experimental data points for CDW (g/L), lipase activity (U/mL), and IZD (mm), respectively. The linear alignment of points along the reference line indicates normal distribution of residuals. (**d**–**f**) Plots of externally studentized residuals versus predicted values for CDW, lipase activity, and IZD, respectively. The random scatter of points within the ±4 standardized boundaries demonstrate homoscedasticity and validates the model’s assumptions. The absence of systematic patterns in residual plots confirms the model’s adequacy in predicting experimental responses.

The normal probability plots (Fig. 5a–c) show that the residuals generally follow a linear trend along the normal probability line, indicating normally distributed errors. The residuals versus predicted plots (Fig. 5d-f) demonstrate random scatter patterns within the standardized boundaries of ± 4, suggesting homogeneous variance. The ANOVA results confirm the model’s significance with high F-values and strong predictive capability with R² values combined with the plot patterns and significant p-values (all < 0.01), validate the model’s adequacy in predicting the experimental responses. ANOVA revealed significant models for cell dry weight (g), lipase activity (U/mL), Inhibition zone diameter (mm), total protein (mg/g) and specific lipase activity (U/mg/g) as follows:

- Cell Dry Weight: Y_CDW_ = + 3.00–0.1578 A + 0.3089B + 0.0932 C + 0.3750AB + 0.2750AC + 0.0250BC + 0.1214 A^2^ – 0.3382B^2^ + 0.0155 C^2^.
- Lipase Activity: Y_Lipase activity_ = 1314.16 + 129.95 A – 81.91B – 8.60 C – 33.38AB + 27.12AC – 39.13BC – 255.95 A^2^– 246.58B^2^ – 307.88 C^2^.
- Inhibition Zone Diameter: Y_IZD _= 4.81 + 0.4150 A – 0.3255B – 0.1108 C + 0.0875AB + 0.1875AC – 0.2375BC – 0.9595 A^2^– 0.9419B^2^ – 1.15 C^2^.
- Protein Content: Y_protein content_ = 6.28–0.3794 A – 0.6173B + 0.2958 C + 0.1928AB – 0.2943AC – 0.0541BC – 0.0769 A^2^+ 0.0328B^2^ + 0.2408 C^2^.
- Specific Lipase Activity: Y_lipase specific activity_ = 210.23 + 24.87 A – 4.96B – 4.17 C – 4.59AB + 5.34AC – 5.07BC – 39.53 A^2^− 39.78B^2^− 51.01 C^2^.

**Table 1:** Observed and predicted CDW, lipase activity, IZD, protein, and specific activity of *B. subtilis* post-CCD optimization.

Run Order	CDW (g/L)		Lipase activity (U/mL)		IZD (mm)		Protein content (mg/mL)		Specific activity (U/mg)	
	Actual Value	Predicted Value	Actual Value	Predicted Value	Actual Value	Predicted Value	Actual Value	Predicted Value	Actual Value	Predicted Value
1	3	3.29	338	397	14	14	5.3	5.8	62.9	69.2
2	1.4	1.62	640	753	24	26	7.24	7.4	88.5	106.0
3	2.8	3.00	1318	1314	47	48	6.1	6.4	214.7	210.2
4	2.8	2.99	436	471	16	17	6.8	5.9	63.5	75.4
5	3	3.00	1318	1314	49	48	6.2	6.4	210.2	210.2
6	2.8	3.05	429	422	17	16	8.6	7.7	49.5	50.9
7	2.6	2.68	560	813	20	28	5.8	5.7	95.7	140.2
8	4	3.71	490	370	19	13	6.3	7.0	77.7	56.5
9	3.2	2.81	335	433	13	14	7.0	6.9	47.2	58.9
10	3	3.00	1318	1314	49	48	6.6	6.4	198.7	210.2
11	3	3.00	1318	1314	48	48	6.48	6.4	203.1	210.2
12	2.4	2.25	961	807	30	27	6.79	6.9	143.3	120.5
13	3	2.76	395	424	16	19	7.1	7.1	55.0	59.8
14	3	3.00	1318	1314	47	48	5.4	6.4	241.8	210.2
15	2.6	2.17	463	483	16	17	5.5	5.3	83.5	89.3
16	3.2	3.00	1318	1314	49	48	6.6	6.4	197.1	210.2
17	2.4	2.68	209	250	0	4	6.8	6.4	30.3	40.0
18	3	2.88	628	539	23	21	5.3	5.1	117.0	99.1
19	2	1.85	825	688	27	20	6.1	6.3	134.3	108.1
20	2	2.37	639	514	27	21	5.4	5.7	112.6	91.3
ANOVA analysis	CDW		Lipase		IZD		Total protein		Specific activity	
F-value	4.71		19.4		18.92		5.03		13.33	
P-value	0.0019		Less than 0.0001		Less than 0.0001		0.0094		0.0002	
R^2^	0.80		0.94		0.94		0.81		0.92	
Model significance	Significant		significant		Significant		Significant		significant	
Lack of fit	Not significant		Not significant		Not significant		Not significant		Not significant	

### Partial purification of lipases produced by *B. subtilis*

The results of the purification procedure are summarized in Table 2. 40–80% ammonium sulfate saturation fractions were tested for lipases precipitation, and 70% showed to have the best effect on precipitating the produced lipases. Ammonium sulfate increased the activity by 2-fold (2628.2 U/mL), while dialysis increased the activity by 5-fold (6570 U/mL).

**Table 2:** Partial purification of produced lipases from *B. subtilis* grown on whey medium using ammonium sulfate assay.

Purification step	Lipases activity (U/ml)	Total protein (mg/mL)	Specific activity (U/mg)	Purification fold
Crude extract	1314 ± 6 ^c^	0.85 ± 0.12 ^c^	1554.1 ± 1.3 ^c^	-
70% Ammonium sulphate fraction	2628.2 ± 1.3 ^b^	0.70 ± 0.05 ^b^	3754.5 ± 1.5 ^b^	2
Dialysis	6570 ± 5 ^a^	0.50 ± 0.08 ^a^	12882.3 ± 5.6 ^a^	5

Specific activity (U/mg) of the sample was calculated by dividing the enzyme units (U) on the total protein content. Values are presented as means ± standard deviation (SD). Different small letters (a, b, c) indicate significant differences (*p* < 0.05) between purification steps based on statistical analysis.

### Biological activities of PPL

#### Antibacterial efficacy and bactericidal mechanism of PPL from *B. subtilis* against *S. aureus*

The inhibitory activity of PPL against *S. aureus* was evaluated using the well diffusion method, showing a significant IZD (mm) of 49 mm, which surpassed those of commercial antibiotics (**Table S3**) like ciprofloxacin (5 mg/mL) and doxycycline (30 mg/mL), with IZDs of 35.4 mm and 29 mm, respectively. Tetracycline (10 mg/mL) and gentamicin (10 mg/mL) displayed moderate activity, while the strain was resistant to ampicillin and azithromycin. Further analysis revealed that the minimum inhibitory concentration (MIC) and minimal bactericidal concentration (MBC) of PPL produced by *B. subtilis* against *S. aureus* strain (Table 3) were 1/8 and 0–1/4 in a serial dilution (0–1/16), respectively, with no antibacterial effect at 1/8 and 1/16 concentrations. The MBC/MIC ratio ≤ 2 confirms PPL’s bactericidal effect against *S. aureus*.

**Table 3:** Minimum inhibitory concentration (MIC), minimal bactericidal concentration (MBC), and mode of action of PPL produced by *B. subtilis* against *S. aureus.*

Pathogenic strain	MIC calculations Inhibition zone (mm)				
	Conc. (mg/mL)				
	PPL	1/2	¼	1/8	1/16
*S. aureus*	54 ± 52^a^	19.0 ± 3.0^b^	12.05 ± 10^c^	0.00	0.00
Spectrum activity (%)	PPL	1/2	1\4	1/8	1/16
	100	100	100	65.7	0
MBC calculations Log_10_ (CFU/mL)					
Conc. (mg/mL)					
Pathogenic strain	PPL	1/2	¼	1/8	1/16
*S. aureus*	–	−	–	+	+
Spectrum activity (%)	3/3	3/3	3/3	0/3	0/3
	100	100	100	0	0
Mode of action	MIC	MBC	MIC/MBC ratio	Mode of action	
*S. aureus*	1/16	1/8	0.5	Bactericidal effect	

Different small letters (a, b, c) indicate significant differences (*p* < 0.05) between different MIC concentrations.

### Biofilm formation and SEM characterization

**Fig. 6:**
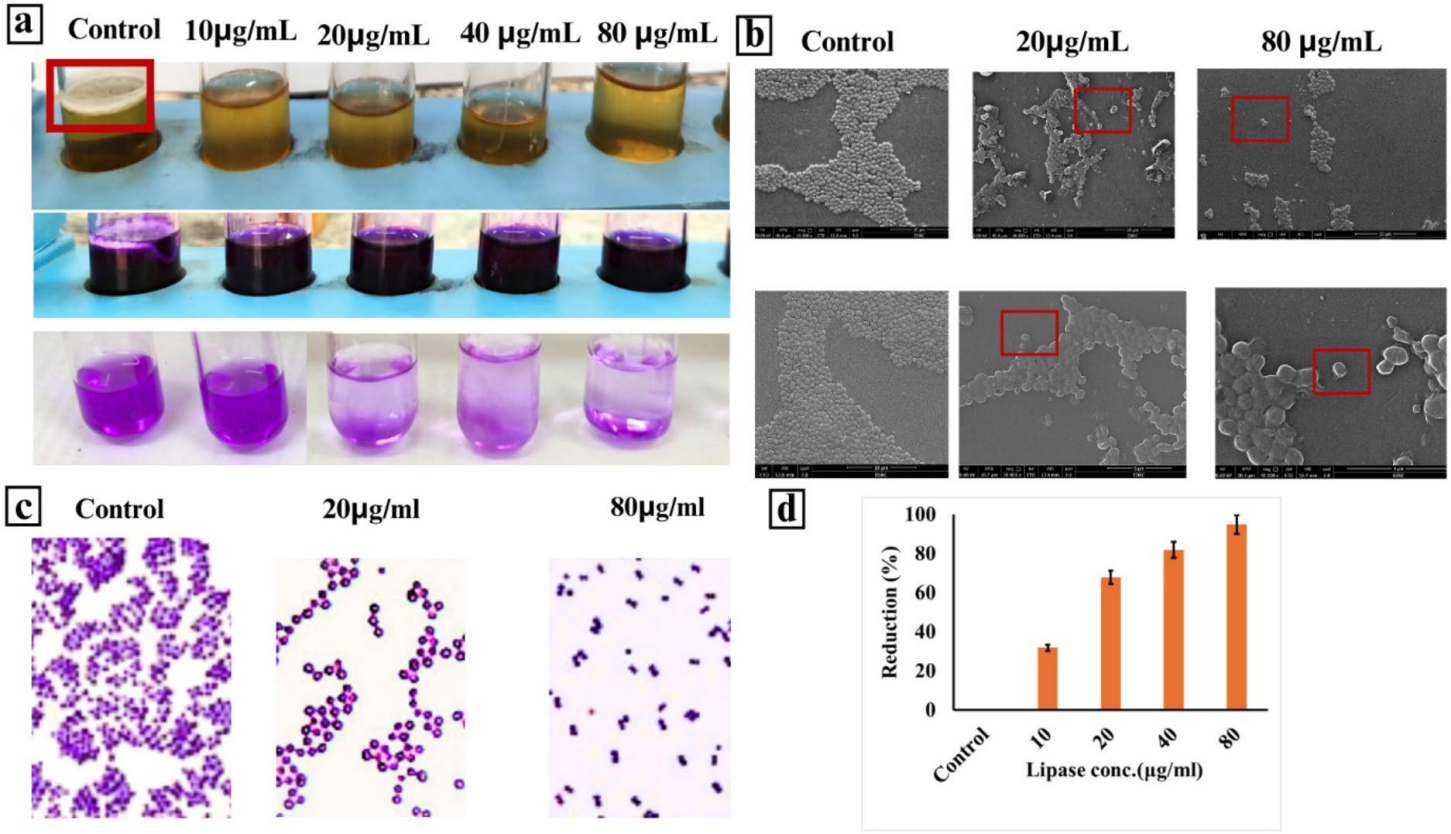
Biofilm inhibition and Scanning electron microscopy images illustrating the effects of the PPL on *S. aureus* cell morphology. (**a**) Untreated control cells show intact cell surfaces. (**b**) SEM analysis for cells treated with 20 and 80 μg/ml PPL display disrupted cell membranes and cell lysis (squared). (**c**) Gram stain for treatments with 20 and 80 μg/ml PPL results in severe membrane damage and cell lysis .

This study investigated the effect of PPL on mature *S. aureus* biofilms that were pre-established for 48 h. Using multiple visualization techniques (light microscopy, SEM) and quantitative analysis, the research demonstrated a significant dose-dependent reduction in biofilm biomass across different PPL concentrations (10, 20, 40, and 80 µg/mL). The highest concentration (80 µg/mL) achieved a 95% reduction in biofilm mass, while the lowest (10 µg/mL) resulted in a 32% reduction, with intermediate concentrations showing proportional effects (*P* < 0.05). The SEM images revealed how PPL treatment disrupted the biofilm architecture, particularly at 20 and 80 µg/mL, showing reduced bacterial cell numbers and disrupted cell-to-cell connections compared to the untreated control. These findings were further supported by light microscopy observations, demonstrating PPL’s ability to penetrate and effectively disrupt the biofilm matrix, visible in both the physical samples (showing color changes in the solutions) and microscopic analyses (revealing structural disruption of the biofilm architecture)., as shown in Fig. 6.

### Time kill assay

**Fig. 7:**
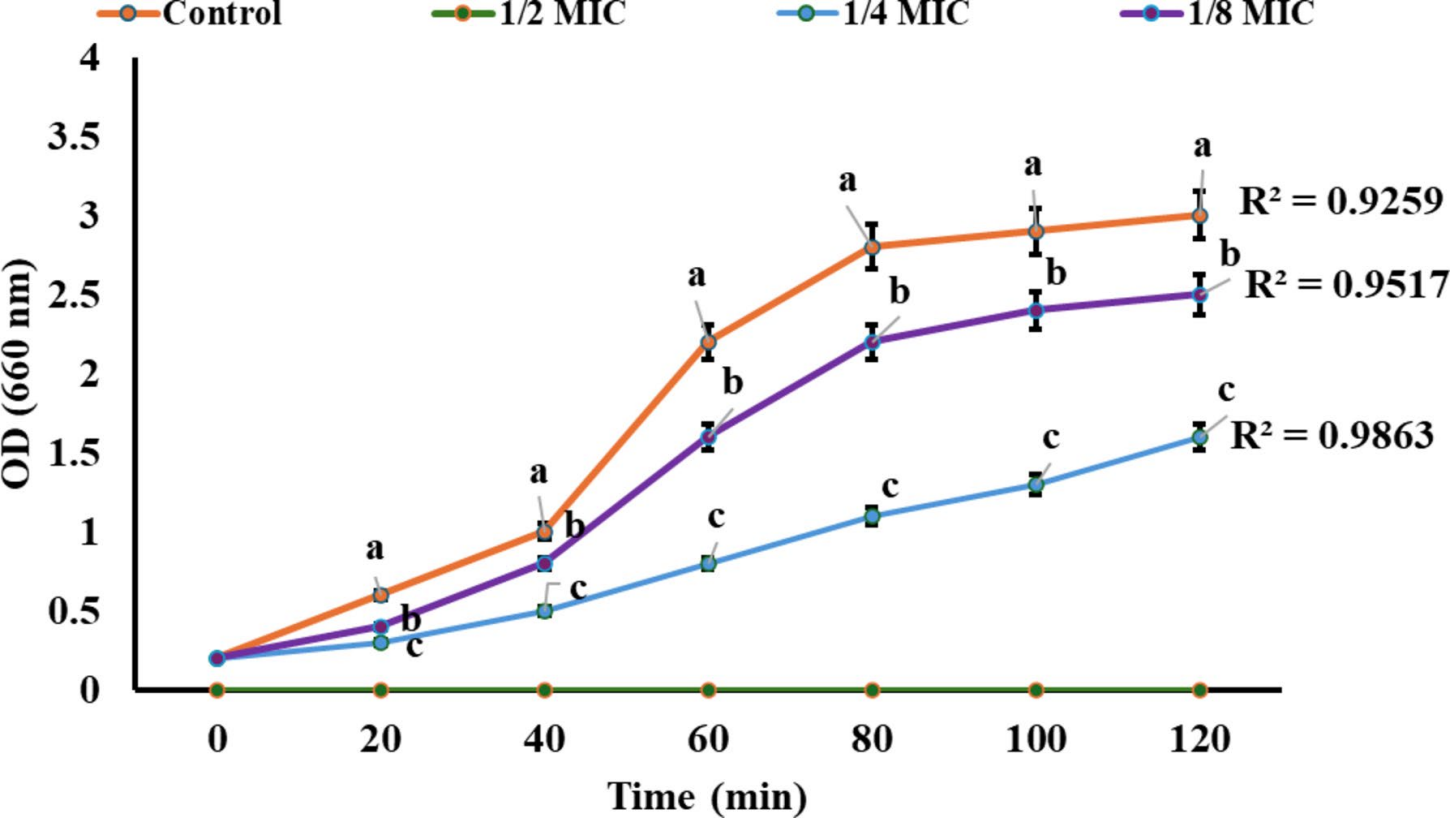
Time-kill kinetics of the PPL against bacterial cells at different multiples of the minimum inhibitory concentration (MIC). Different small letters (**a**,** b**,** c**) indicate significant differences (*p < 0.05*) between different MIC concentrations.

The growth of *S. aureus* strain was monitored at OD 600 nm under different sub-MIC concentrations of PPL (1/8, 1/4, and 1/2 MIC) and compared to an untreated control over 120 min. The growth curves demonstrate a clear concentration-dependent inhibitory effect of PPL. The control strain showed normal growth progression, reaching an OD of approximately 3.0 (R² = 0.9656). At 1/8 MIC, there was a moderate reduction in growth, with the OD reaching about 2.8 (R² = 0.9728), while 1/4 MIC showed more substantial inhibition with the OD only reaching approximately 1.8 (R² = 0.9782). The most dramatic effect was observed at 1/2 MIC, where bacterial growth was completely inhibited, maintaining baseline OD values throughout the observation period. These results demonstrate that PPL exhibits significant concentration-dependent antibacterial activity even at sub-MIC levels, with increasing concentrations leading to progressively stronger growth inhibition of the *S. aureus* strain as shown in Fig. 7.

#### Virulence Attenuation after PPL treatment

**Fig. 8:**
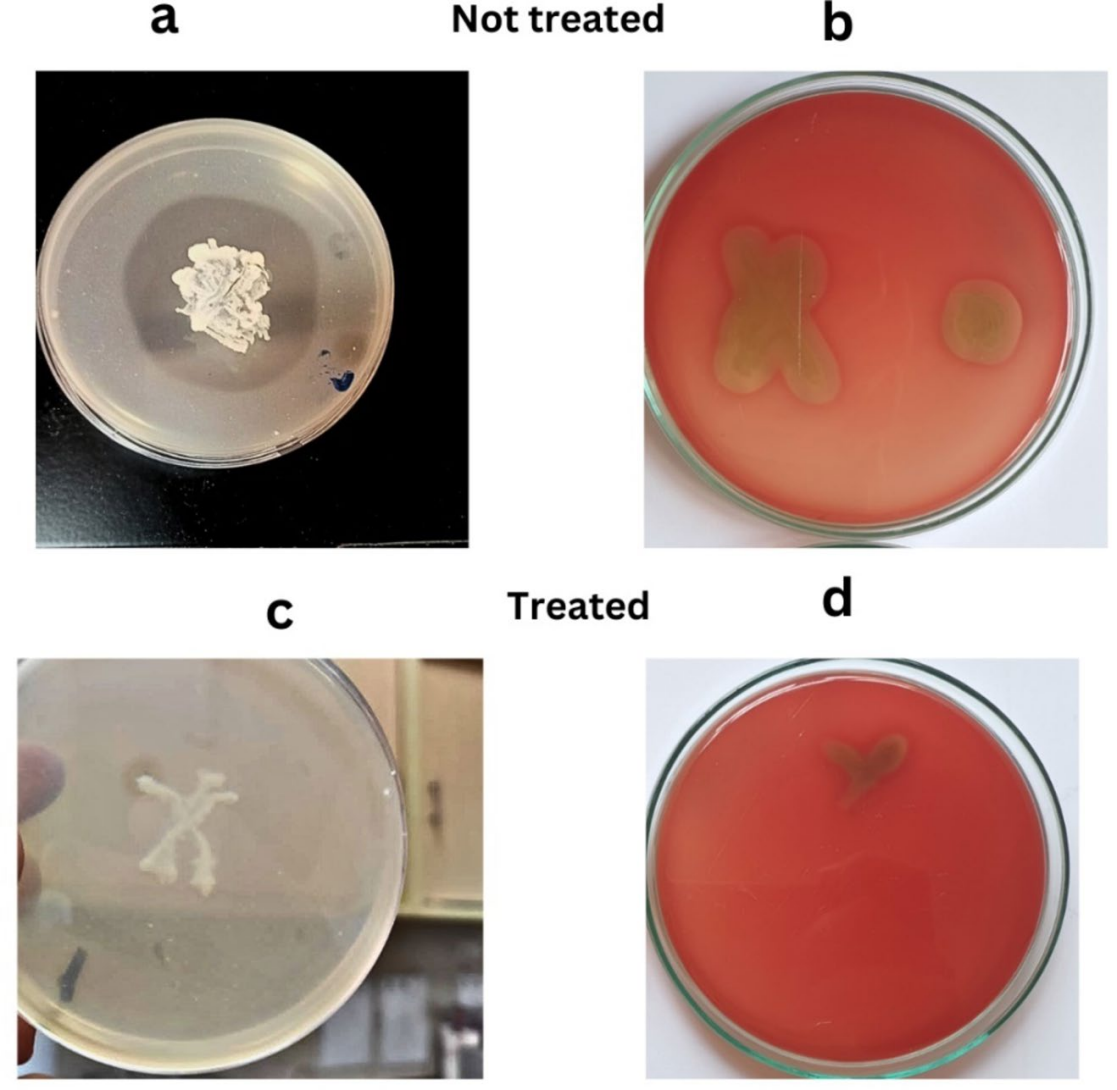
Impact of PPL treatment on hemolysin and protease production by *B. subtilis* against *S. aureus*. (**a**) Untreated control cells showing normal growth on skim milk agar plates. (**b**) Characteristic clearing zone around bacterial colony on blood agar, indicating hemolysin production in untreated cells. (**c**)Treated cells exhibiting reduced growth and diminished proteolytic activity, as evident from the lack of a clear zone. (**d**) Treated cells show decreased hemolytic activity, with smaller and fainter zones of clearance compared to the untreated control in (**b**).

As shown in Fig. 8, PPL showed high proteolytic and hemolytic against *S. aureus* strain as it was significantly (*P* < *0.05*) inhibited by (25–100%) and (18.8–63.6%) for 4 and 2 µg/ml of PPL respectively. Untreated *S. aureus* strain showed a visible clear zone detected around the bacterial growth, while PPL inhibits hemolytic activity, and no visible clear zone was detected around the bacterial growth.

### Cytotoxicity of produced PPL against normal liver cell line

**Fig. 9:**
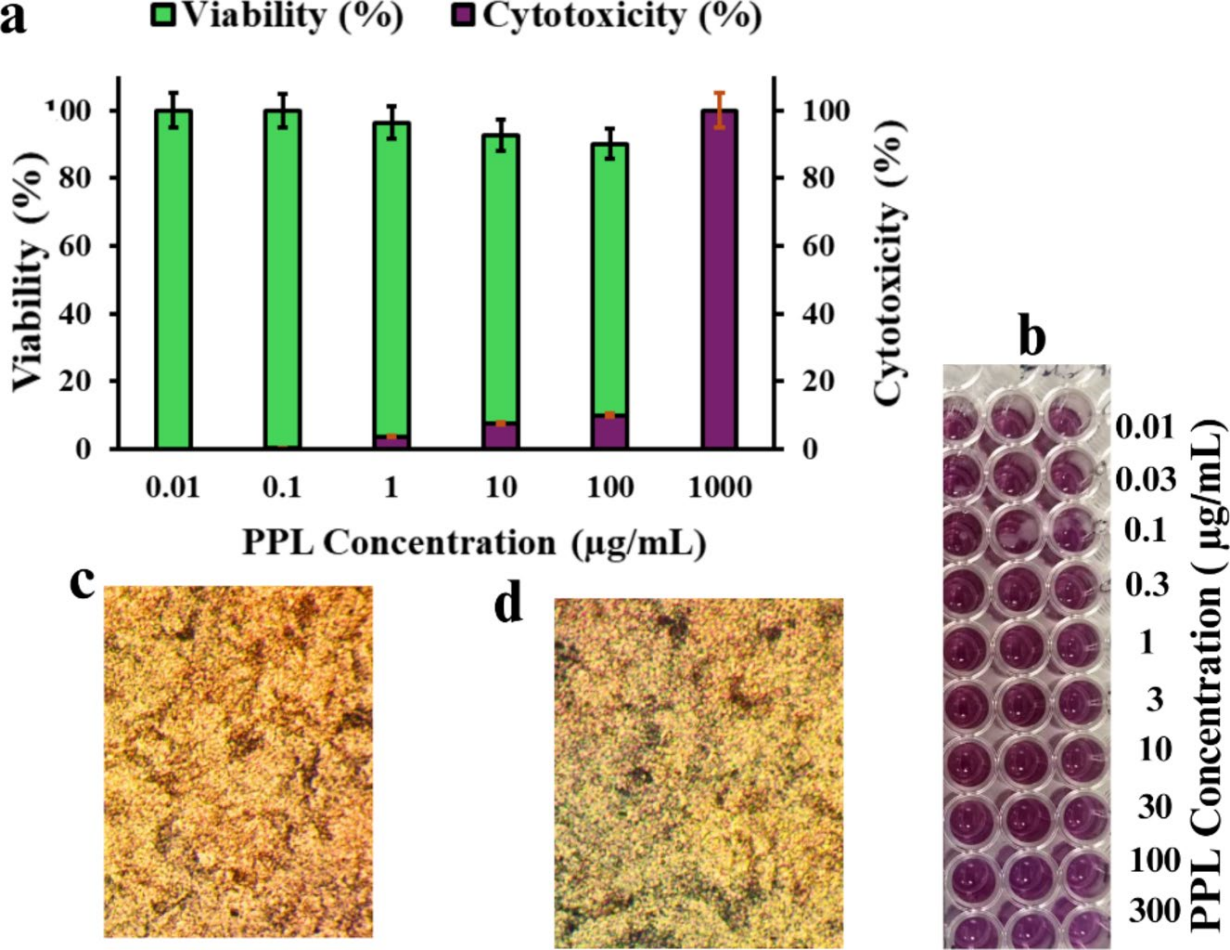
Cytotoxicity of partially purified lipase (PPL) from *B. subtilis* on normal mouse liver cells. (**a**) Cell viability and cytotoxicity (%) of normal mouse liver cells treated with varying concentrations of PPL (0.01–1000 µg/mL), with IC50 value >300 μg/mL indicating low cytotoxicity. (**b**) Representative 96-well plate showing the colorimetric response for different PPL concentrations (0.01–300 µg/mL). **(c**–**d)** Microscopic images showing cellular morphology after treatment with increasing concentrations of PPL. **(c)** Control group and **(d)** Moderate concentration.

As illustrated in Fig. 9, PPL from *B. subtilis* was tested for cytotoxicity against the mouse normal liver cell line. It was tested at concentrations ranging from 0.01 µg/mL to 300 µg/mL. Cell viability was measured to determine the IC50 value. At the highest tested concentration of 300 µg/mL, P1 treatment resulted in 68–71% cell viability. The IC50 value for cytotoxicity of the PPL against normal mouse liver cells was greater than 300 µg/mL. Therefore, the PPL sample showed low toxicity against the normal liver cell line, with an IC50 exceeding the maximum tested concentration of 300 µg/ml. Over two-thirds of the liver cells survived treatment even at this high 300 µg/mL dose.

### GC/MS for the produced metabolites by *B. subtilis*

The analysis in Table 4 shows significant changes in fatty acid composition between the pre-degradation and post-degradation samples. Initially, the oil contained primarily oleic acid 3-hydroxypropyl ester (22.80 area), followed by octadecane derivative (3.25 area), linoleic acid (2.04 area), stearic acid (1.29 area), and smaller amounts of palmitic acid (0.24 area) and oleic/11-octadecenoic acid (0.17 area each). After degradation, the profile shifted dramatically, with increased presence of medium to long-chain fatty acids: myristic acid (4.96 area), palmitic acid (4.59 area), linoleic acid (increased to 5.04 area), and oleic acid (4.89 area). Notably, the 3-hydroxypropyl ester of oleic acid and the octadecane derivative were completely absent in the degraded sample, suggesting substantial biochemical transformation of the waste oil during lipase production by *Bacillus subtilis*, with apparent hydrolysis of esters and enrichment of free fatty acids.

**Table 4:**
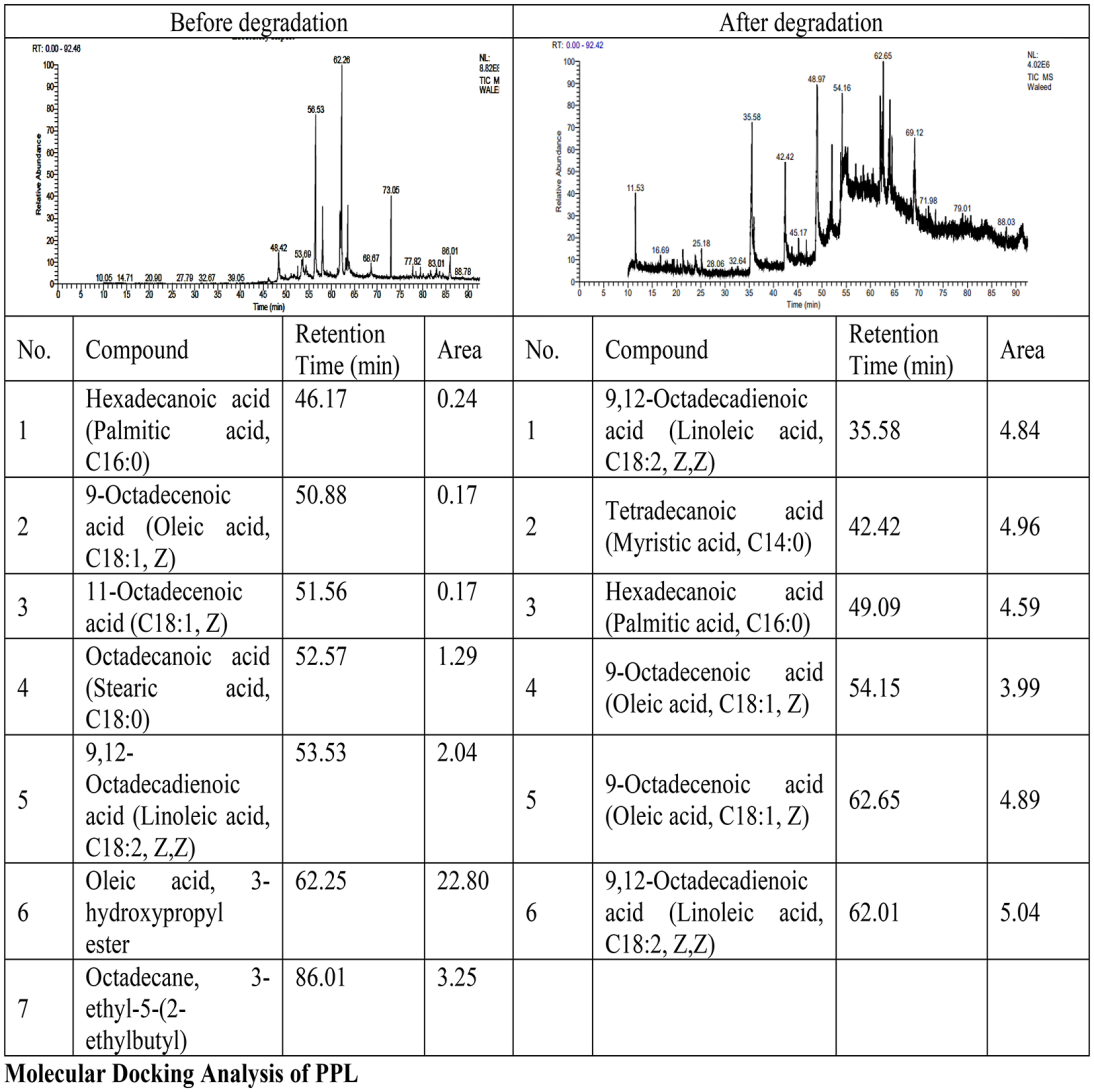
GC/MS analysis for frying oil waste and *B. subtilis* culture grown on whey medium supplemented with 2% frying oil waste.

#### Molecular Docking analysis of PPL

**Fig. 10:**
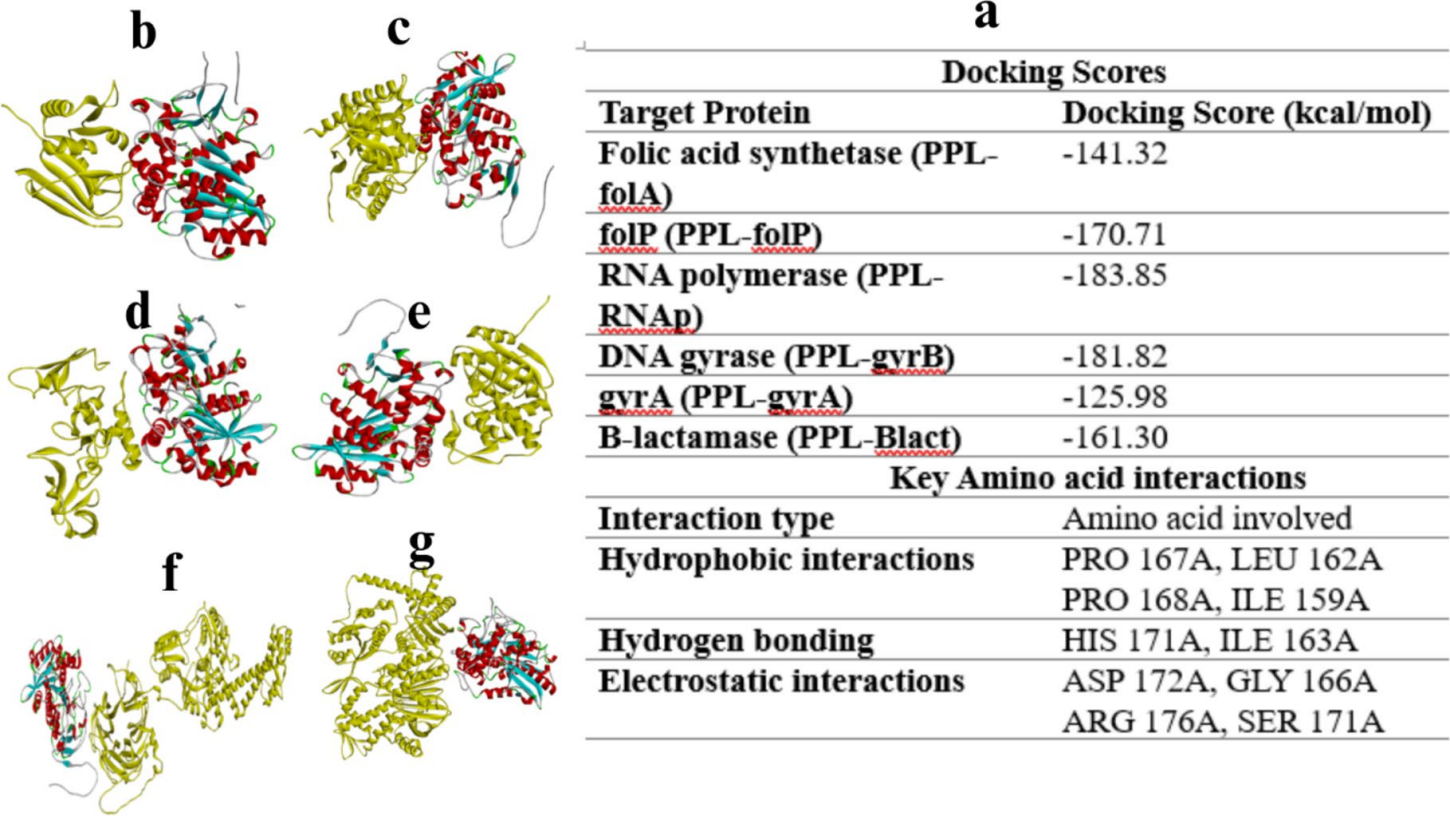
Molecular docking for PPL interaction with *S. aureus* various bacterial protein targets.

Figure 10 highlight the inhibitory effects of PPL on bacterial enzymes essential for survival, including folic acid synthetase, RNA polymerase, and DNA gyrase, leading to inhibition of DNA synthesis and microbial cell death. PPL also disrupts cell wall synthesis by inhibiting β-lactamase. Molecular docking analysis reveals key interactions stabilizing these effects, such as hydrophobic interactions between PRO 167 A and LEU 162 A, and PHE 168 A with ILE 159 A, along with hydrogen bonding between HIS 171 A and ILE 163 A, and electrostatic interactions involving ASP 172 A and GLY 166 A, as well as ARG 176 A with SER 171 A. Docking scores show strong binding affinities, with notable values such as -141.32 for folic acid synthetase (PPL-folA), -170.71 for PPL-folP, -183.85 for RNA polymerase (PPL-RNAp), -181.82 for DNA gyrase (PPL-gryB), -125.98 for gryA, and − 161.30 for β-lactamase (PPL-Blact). These scores emphasize PPL’s potent interactions with bacterial targets.

The docking scores represent the binding affinities calculated from computational simulations between the PPL enzyme and different bacterial proteins involved in folate metabolism a: (folA, b: folP), c: RNA polymerase (RNAp), d: DNA gyrase (d: gyrB, e: gyrA), and f: β-lactamase (Blact). Lower (more negative) docking scores indicate stronger predicted binding interactions. The data suggests that PPL exhibits the highest binding affinity for RNAp (-183.85), followed by gyrB (-181.82) and folP (-170.71), potentially conferring antimicrobial activity by targeting these essential bacterial proteins.

## Discussion

Lipases are essential enzymes with wide-ranging applications in industrial, pharmaceutical, and biotechnological sectors, owing to their ability to hydrolyze lipids efficiently^26^. Among various microbial sources, *B. subtilis* has emerged as a promising candidate for lipase production due to its resilient growth, metabolic versatility, and ability to produce extracellular enzymes^27^. This study investigates the optimization, purification, and characterization of lipase produced by *B. subtilis*, emphasizing its antibacterial and antibiofilm properties against *S. aureus*. The findings provide valuable insights into enzyme production kinetics, key influencing factors, and the therapeutic potential of lipase-derived products.

This study demonstrates that *B. subtilis* growth rate, lipase activity, and inhibitory potential are significantly influenced by incubation time, with optimal performance occurring at 48 h and 30 °C. This aligns with previous research, which shows maximum enzyme activity and bacterial growth during the mid-logarithmic phase, when cells are most metabolically active. The observed growth rate and enzyme production kinetics are typical for *B. subtilis* under optimal conditions. Lipase production is growth-associated, with peak activity during the exponential phase and a decline as the culture enters stationery and death phases, likely due to nutrient depletion, metabolic by-products, or enzyme degradation^15^. Strong correlations between incubation time and bacterial growth, lipase activity highlight the interconnection of these processes during the mid-logarithmic phase^28^. Our results are consistent with prior studies showing that *B. subtilis* strains exhibit optimal activity at intermediate incubation periods, with declines attributed to nutrient exhaustion and environmental stress^29^.

The optimization of lipase production by *B. subtilis* using Response Surface Methodology (RSM), through Plackett–Burman Design (PBD) and Central Composite Design (CCD), led to significant improvements in enzyme yield and anti-staphylococcal activity. PBD screening identified whey, peptone, and agitation speed as key factors influencing lipase production, consistent with previous studies highlighting the importance of nitrogen and carbon sources like whey (a rich carbon source) and peptone (providing essential amino acids for growth and enzyme synthesis)^30^. Agitation speed is crucial for proper oxygen transfer and nutrient distribution, essential for microbial enzyme activity. The optimization through CCD resulted in robust statistical models with high R² values (> 0.90) and significant F-values (*P* < 0.01), confirming the predictive power of the models. Notable interaction effects were observed between whey and peptone, as well as between peptone and agitation speed, indicating their synergistic roles in lipase synthesis^8^.

The partial purification of lipases produced by *B. subtilis* demonstrated significant increases in enzyme activity. The 70% ammonium sulfate saturation fraction resulted in a 2-fold increase in lipase activity (2628.2 U/ml) compared to the crude extract (1314 U/ml). This increase aligns with studies suggesting that ammonium sulfate precipitation works by altering protein solubility, leading to precipitation, and the high ionic strength of ammonium sulfate reduces protein-protein repulsion, promoting aggregation and precipitation at optimal saturation levels, typically around 70% ^17^. Following ammonium sulfate precipitation, the dialysis step led to a substantial 5-fold increase in lipase activity (6570 U/ml), likely due to the removal of small molecular weight contaminants, such as salts and impurities that could inhibit enzyme activity, resulting in a more purified enzyme preparation with a final specific activity of 12882.3 U/mg^31^.

This study demonstrates the potent antibacterial properties of PPL derived from *B. subtilis* against *S. aureus*, particularly multidrug-resistant strains. The inhibition zone diameter (IZD) of 49 mm achieved by PPL significantly outperformed several commercially available antibiotics, such as ciprofloxacin (35.4 mm) and doxycycline (29 mm), indicating superior antibacterial efficacy. The MIC (1/16 mg/mL) and MBC (1/8 mg/mL) values suggest a bactericidal effect, reinforced by the MBC/MIC ratio (≤ 2), which is consistent with the bactericidal nature of *B. subtilis*-derived antimicrobial peptides, including subtilosin. These peptides disrupt bacterial cell membranes, leading to cellular leakage and death, supporting the observed effects of PPL^32^. Additionally, PPL demonstrated strong biofilm inhibition, with a 95% reduction in biofilm biomass at the highest concentration (80 µg/mL), a key finding given the challenge of treating *S. aureus* infections associated with biofilm formation. Biofilms protect bacteria from host immune responses and antibiotics, making infections difficult to treat. PPL’s ability to penetrate and disrupt the biofilm matrix aligns with research on antimicrobial agents from *Bacillus spp.*, which interfere with bacterial adhesion and communication, thereby disrupting biofilm integrity^5^.

The observed progressive increase in growth inhibition with PPL concentration, culminating in complete growth inhibition at 1/2 MIC, highlights the compound’s potential to destabilize bacterial membranes and biofilm matrices. This is likely due to cumulative phospholipid hydrolysis, a mechanism known for destabilizing bacterial cell membranes and promoting lysis. Lipases from *Bacillus* species are known to hydrolyze phospholipids and fatty acids, which disrupt bacterial membranes and contribute to cell death. Additionally, lipase activity likely aids in degrading lipid-based components in the extracellular polymeric substances (EPS) of biofilms, weakening the biofilm matrix and enhancing antimicrobial efficacy^33^. This action, combined with lipase interference in bacterial quorum sensing pathways, further reduces bacterial pathogenicity, contributing to the overall therapeutic potential of PPL^5^.

Lipase treatment significantly attenuates the virulence of *S. aureus*, as shown by reductions in both proteolytic and hemolytic activities. The inhibition of proteolytic activity ranged from 25 to 100%, and hemolysin activity was inhibited by 18.8–63.6% at concentrations of 4 and 2 µg/ml, respectively. These findings suggest that PPL interferes with the production of key virulence factors in *S. aureus*, including proteases and hemolysins, which are essential for tissue degradation and immune evasion. Proteases in *S. aureus* contribute to host tissue degradation and immune system avoidance, and their inhibition by PPL likely reduces the bacterium’s capacity to damage host tissues^34^. This aligns with previous studies showing the crucial role of proteases in *S. aureus* pathogenesis. Many studies revealed that electron microscopy studies of red blood cells after lipases treatment revealed lysis, suggesting that the reduction in hemolysin production is associated with the bacterium’s diminished ability to lyse host cells^35^ which is consistent with research showing that protease and phospholipase inhibitors can decrease the hemolytic activity of *S. aureus*.

The cytotoxicity of partially purified lipase (PPL) produced from *B. subtilis* was evaluated using normal mouse liver cells, with the results showing that PPL exhibits low toxicity, even at high concentrations. At 300 µg/ml, PPL treatment resulted in 68–71% cell viability, and the IC50 value for cytotoxicity was found to be greater than 300 µg/ml, indicating that PPL does not induce significant cytotoxic effects on liver cells. The high cell viability at the maximum concentration suggests that PPL does not adversely affect normal liver cells, making it a promising candidate for therapeutic applications with minimal risk of off-target toxicity. These findings are consistent with previous studies showing that bacterial lipases and proteases generally exhibit low cytotoxicity against mammalian cells, with no significant cell death observed at concentrations typically used in industrial or therapeutic settings^36^.

Some studies have reported that certain lipases exhibit selective cytotoxicity against cancer cell lines. For example, microbial lipases, particularly those derived from *Pseudomonas* species, have shown cytotoxic effects on human breast cancer (MCF-7) and colorectal cancer (HCT-116) cells by inducing apoptosis and disrupting lipid metabolism^37^. Additionally, lipases from Aspergillus species have been observed to generate cytotoxic effects through the production of free fatty acids, leading to oxidative stress and cell membrane damage in glioblastoma (U87) cells^38^. Contrary to their potential anticancer effects, some lipases have demonstrated toxicity in normal cell lines. Studies on human fibroblasts (HSF) and normal epithelial cells have reported that high concentrations of bacterial lipases can disrupt membrane integrity due to excessive hydrolysis of phospholipids^39^. In human kidney (HEK-293) and liver (HepG2) cell lines, prolonged exposure to certain lipases led to increased ROS (reactive oxygen species) production, mitochondrial dysfunction, and reduced cell viability, particularly when derived from pathogenic bacterial strains such as Staphylococcus aureus^40^. The cytotoxicity of lipase appears to be dose-dependent. Low concentrations often do not cause significant harm and may even support cell growth by facilitating lipid metabolism. However, higher concentrations (> 100 µg/mL) tend to induce apoptosis or necrosis in both cancerous and non-cancerous cells, suggesting that excessive lipid hydrolysis can disrupt cellular homeostasis^41^. This effect is particularly pronounced in lipid-rich tissues, such as hepatocytes, where lipase overactivity may lead to lipotoxicity.

Mechanistically, lipase-induced cytotoxicity has been linked to: Excessive lipid hydrolysis can break down essential membrane components, leading to cell lysis. The release of free fatty acids can result in increased ROS levels, which damage proteins, lipids, and DNA. Some studies indicate that lipases trigger mitochondrial membrane permeabilization, leading to apoptosis. Lipase-treated cells often exhibit increased pro-inflammatory cytokine expression, suggesting an immune response activation^42^. The cytotoxicity of lipase is highly context-dependent. While some lipases have promising anticancer properties, others pose risks to normal cells, especially at high doses. Further studies are needed to explore strategies for controlling lipase activity to maximize therapeutic benefits while minimizing cytotoxicity. Future research should focus on enzyme modifications, selective targeting, and controlled release mechanisms to harness lipase applications safely in biomedical fields.

The GC/MS analysis of fatty acids produced by *B. subtilis* grown on whey supplemented with frying oil waste revealed the presence of both saturated and unsaturated fatty acids, highlighting the bacterium’s broad substrate specificity for lipase production and lipid metabolism. A key finding was the detection of 9,12-Octadecadienoic acid (Z, Z), a known lipase inducer, which suggests its role in stimulating lipase production. This aligns with previous studies that show long-chain unsaturated fatty acids induce lipase gene expression in *B. subtilis*^43^. This indicates that *B. subtilis* metabolizes fatty acids from the frying oil waste to enhance lipase production. In addition to unsaturated fatty acids, significant levels of saturated fatty acids, including Tetradecanoic acid and Hexadecanoic acid, were detected. These are utilized by *B. subtilis* as energy sources, further demonstrating the bacterium’s metabolic flexibility and ability to grow and produce lipase from complex substrates like frying oil waste^44^. The presence of Oleic acid (9-Octadecenoic acid (Z)) at two retention times also confirms efficient utilization of frying oil waste, with Oleic acid known to enhance lipase production. Additionally, the disappearance of octadecane derivatives highlights the potential role of *B. subtilis* in degrading hydrocarbon contaminants, further supporting its biodegradative efficiency^45^.

GC/MS analysis identified various fatty acids, which have been previously documented for their antibiofilm properties against *S. aureus* at low concentrations. Notable examples include oleic acid (18:1 ω-9, cis) at 100 µg/mL, linoleic acid (18:2 ω-6) at 20 µg/mL, cis-11-eicosenoic acid (20:1 ω-9) and cis-11,14-eicosadienoic acid (20:2 ω-9) at 10 µg/mL, as well as docosahexaenoic acid (DHA; C22:6, ω-3) and eicosapentaenoic acid (EPA; C20:5, ω-3) at 20 µg/mL. Additionally, myristoleic acid (14:1, ω-5) at 2 µg/mL was found to inhibit *S. aureus* biofilm formation^46^. In the current study, oleic acid (18:1 ω-9, cis) demonstrated significant inhibition of *S. aureus* biofilm development. These findings indicate that fplaysrs such as carbon chain length, degree of unsaturation, and the position and configuration of double bonds play a crucial role in antibiofilm activity against *S. aureus*^46^.

The molecular docking analysis of PPL demonstrates its potential as a potent antimicrobial agent by inhibiting critical bacterial enzymes involved in DNA synthesis and cell wall formation. PPL interacts strongly with several bacterial targets, including folic acid synthetase, RNA polymerase, DNA gyrase, and β-lactamase, all of which are essential for bacterial survival. This suggests that PPL could serve as a broad-spectrum inhibitor, disrupting key bacterial biochemical pathways. A key interaction was observed between PPL and RNA polymerase (PPL-RNAp), with the strongest binding affinity, exhibiting a docking score of -183.85. RNA polymerase is essential for bacterial DNA transcription, and its inhibition halts protein synthesis and bacterial growth^47^. This result aligns with previous studies showing that targeting RNA polymerase effectively inhibits bacterial replication, suggesting PPL’s significant antimicrobial potential^48^. PPL also demonstrated strong binding to DNA gyrase, with docking scores of -181.82 for gyrB and − 125.98 for gyrA. DNA gyrase is crucial for bacterial DNA supercoiling during replication, and its inhibition leads to DNA accumulation and bacterial cell death^49^. The strong interaction between PPL and DNA gyrase further supports its potential in disrupting bacterial DNA replication^50^. Additional interactions were seen between PPL and folic acid synthetase (PPL-folA) and folate reductase (PPL-folP), with docking scores of -141.32 and − 170.71, respectively. These enzymes are involved in folate synthesis, a key component for bacterial DNA replication. Inhibiting folate metabolism is a well-established antimicrobial strategy^51^, and the observed docking scores suggest that PPL could interfere with folate synthesis, reinforcing its antimicrobial activity. Furthermore, PPL bound to β-lactamase with a docking score of -161.30. β-lactamase hydrolyzes β-lactam antibiotics, rendering them ineffective. Inhibiting β-lactamase by PPL suggests that it could enhance the effectiveness of β-lactam antibiotics, providing an additional mechanism to combat bacterial resistance^52^. Several studies have reported docking scores for well-established antibiotics, providing a benchmark for evaluating PPL’s predicted interactions. For instance, ciprofloxacin and rifampicin, known inhibitors of DNA gyrase and RNA polymerase, exhibit docking scores ranging from − 120 to -160 kcal/mol^53^. In comparison, PPL demonstrated stronger binding affinities, with scores of -181.82 kcal/mol for DNA gyrase and − 183.85 kcal/mol for RNA polymerase. Similarly, β-lactam antibiotics, which inhibit β-lactamase, typically show scores of -130 to -150 kcal/mol, whereas PPL achieved − 161.30 kcal/mol, suggesting a potentially enhanced interaction^54^. These findings imply that PPL may exhibit antimicrobial potential comparable to or greater than established antibiotics. However, computational docking alone cannot confirm biological activity. Enzyme inhibition assays testing is essential to assess PPL’s efficacy and potential as a novel antimicrobial agent.

While this study provides valuable insights into the optimization, purification, and antibacterial activity of PPL from *B. subtilis*, there are limitations. The study was conducted under laboratory conditions, which may not fully replicate real-world environments. Future research should focus on large-scale fermentation processes and in vivo testing to validate the therapeutic efficacy and safety of PPL. Additionally, exploring genetic modifications of *B. subtilis* could enhance lipase yield and bioactivity. Further investigations into the mechanism of action at the molecular level and the potential synergistic effects of PPL with existing antibiotics could open new avenues for combating antibiotic-resistant bacterial infections.

## Conclusion

This study highlights the potential of optimized lipase production from *B. subtilis* as an eco-friendly and effective alternative to traditional antibiotics, especially against resistant pathogens like *S. aureus*. The optimized production process and the enzyme’s efficacy in biofilm disruption and virulence attenuation make it a promising candidate for therapeutic applications. The identification of key fatty acids such as 9,12-Octadecadienoic acid, Tetradecanoic acid, and Oleic acid in cheese whey underscores its potential as a sustainable substrate for antimicrobial lipase production, with each fatty acid’s retention time and peak area supporting its notable presence. The successful use of PBD and CCD further demonstrates the benefits of statistical modeling in optimizing microbial enzyme production, providing a roadmap for future studies on other valuable microbial enzymes.

## Electronic supplementary material

Below is the link to the electronic supplementary material.

Supplementary Material 1

## Data Availability

The raw data and analysed data used during the current study are available from the corresponding author upon reasonable request. All microbial pathogens were provided by the MIRCEN, Faculty of Agriculture, Ain Shams University, Cairo, Egypt, and were deposited in the following strain providers: 1. *Bacillus subtilis* DSM 1088 https://bacdive.dsmz.de/strain/962 2. *Staphylococcus aureus* ATCC 6538 https://www.atcc.org/products/6538.
