# Publisher Correction: Optimizing lipase production by *Bacillus subtilis* on cheese whey and evaluating its antimicrobial, antibiofilm, anti virulence and biosafety properties

**DOI:** 10.1038/s41598-025-00662-7

**Published:** 2025-05-26

**Authors:** Mohamed Y. Abo El-Naga, Muhammad A. Khan, Samah H. Abu-Hussien, Samar M. Mahdy, Ammar AL-Farga, Aml A. Hegazy

**Affiliations:** 1 Food Science Department, Faculty of Agriculture, Ain Shams University, Cairo, 11241 Egypt; 2 Department of Biological Sciences, Faculty of Sciences, International Islamic University (IIU), Islamabad, Pakistan; 3 Agricultural Microbiology Department, Faculty of Agriculture, Ain Shams University, Cairo, 11241 Egypt; 4 Department of Biochemistry, Faculty of Science, University of Jeddah, Jeddah, Saudi Arabia

Correction to: *Scientific Reports* 10.1038/s41598-025-92181-8, published online 01 April 2025

In the original version of the Article, Figure 4 was a duplication of Figure 3.

The original Article has been corrected.

