# Supplementary material for: Optimizing lipase production by Bacillus subtilis on cheese whey and evaluating its antimicrobial, antibiofilm, anti virulence and biosafety properties
Source: Sci Rep. 2025 Apr 1;15:11087. doi: 10.1038/s41598-025-92181-8 (PMC11961612; doi:10.1038/s41598-025-92181-8)
Supplement: Supplementary file 1 — Supplementary Material 1 [file 41598_2025_92181_MOESM1_ESM.pdf]

Supplementary Material

Scientific Reports

Optimizing Lipase production by *Bacillus subtilis* on cheese whey and evaluating its Antimicrobial, Antibiofilm, Anti virulence and biosafety properties

Mohamed Y. Abo El-Naga[1†], Muhammad A. Khan[2*†], Samah H. Abu-Hussien[3*†], Samar M. Mahdy[1], Ammar AL-Farga[4], Aml A. Hegazy[1]

[1]Food Science Department, Faculty of Agriculture, Ain Shams University, Cairo, 11241, Egypt

[2]Department of Biological Sciences, Faculty of Sciences, International Islamic University (IIU), Islamabad, Pakistan

[3]Agricultural Microbiology Department, Faculty of Agriculture, Ain Shams University, Cairo, 11241, Egypt

[4]Department of Biochemistry, Faculty of Science University of Jeddah, Saudi Arabia.

*Corresponding author: Samah H. Abu-Hussien (samah_hashem1@agr.asu.edu.eg)

*Corresponding author: Muhammad A. Khan (muhammadaslamkhanmarwat@gmail.com)

[†]Equal contribution authors

Table S1: Levels of different factors tested in Plackett–Burman design for lipase production by *B. subtilis*

**Factor**	**Unit**	**Symbol**	**Coded levels**	
			**-1 (Low)**	**+1 (High)**
**Frying oil waste**	mL/L	(A)	15	30
**Whey**	mL/L	(B)	20	40
**Tryptone**	g/L	(C)	15	30
**pH**		(D)	6	8
**$MgSO_4$**	g/L	(E)	0.2	2
**Peptone**	g/L	(F)	15	30
**Mannitol**	g/L	(G)	10	20
**Agitation speed**	rpm	(H)	100	250

Table S2: Levels of significant factors tested in the CCD design for lipase production by *B. subtilis*

Factor	Symbol	Levels of the variables tested in CCD	
		-1 (Low)	+1 (High)
**Whey**	(A)	20	40
**Peptone**	(B)	15	30
**Agitation speed**	(C)	100	250

Table S3: Antibiotic susceptibility test against *S. aureus*

Antibiotic	Conc. (mg/mL)	IZD (mm)	Susceptibility mode
Amoxicillin (AX)	10.0	18.5 ± 7.5[f]	I
Ampicillin (AMP)	10.0	0.000 ± 0[g]	R
Azithromycin (AZM)	15.0	0.000 ±0[g]	R
Ciprofloxacin (CIP)	5.00	35.4 ± 6.2[c]	S
Doxycycline (DOX)	30.0	40.0 ± 1.5[b]	S
Norfloxacin (NX)	10.0	29.5 ± 9.2[c]	I
Gentamicin (GEN)	10.0	29.7 ± 1.5[d]	I
Kanamycin (K)	30.0	22.9 ± 1.6[e]	I
Rifampin (RIF)	5.00	28.9 ± 3.1[d]	I
Tetracycline (TE)	10.0	35.9 ± 4.2[b]	I
Crude lipase	100%	54.0± 4.2[a]	S

R: Resistant; I, intermediate; S, sensitive; CLSI, Clinical Laboratory Standards Institute; EUCAST, European Committee on Antimicrobial Susceptibility Testing; BSAC, British Society for Antimicrobial Chemotherapy: NI, No Inhibition. All values are the mean of three replicates ± standard deviation (SD).
